# Effect of Material Property Factors on the Interfacial Residual Stress Gradient in High-Manganese Steel/STS304 Dissimilar Butt Welds

**DOI:** 10.3390/ma19183921

**Published:** 2026-09-15

**Authors:** Jeong-Ung Park, Gyubaek An

**Affiliations:** 1 Department of Civil Engineering, Chosun University, 309 Pilmun-daero, Dong-gu, Gwangju 61452, Republic of Korea; jupark@chosun.ac.kr; 2 Department of Naval Architecture and Ocean Engineering, Chosun University, 309 Pilmun-daero, Dong-gu, Gwangju 61452, Republic of Korea

**Keywords:** high-manganese steel, dissimilar welding, welding residual stress, thermal expansion mismatch, finite element analysis, interfacial stress gradient

## Abstract

Cryogenic high-manganese austenitic steel is used in liquefied natural gas (LNG) storage tanks and has a lower thermal expansion coefficient than other cryogenic steels. Because it is joined to stainless steel in the loading system, dissimilar welding is required. Three-dimensional thermal elastic–plastic finite element analysis and sectioning measurements were performed on 15-mm-thick high-manganese steel/STS304 dissimilar butt welds, and the residual stress was analysed separately in the longitudinal and transverse components. The analysis predicted a steep gradient at the weld metal–STS304 interface 8.5 mm from the weld centre, where the transverse stress falls to about −230 MPa. The longitudinal peak differed from the homogeneous joint by only 77 MPa (−11.7%), within the analysis–measurement deviation (RMSE 103–127 MPa). Of the four properties substituted individually, the expansion coefficient gave the highest reproduction ratio (57.9% on average) and alone reproduced the interfacial compression; substituting all four reproduced the effect almost completely (102.4%). The transverse stress range increased linearly with the expansion mismatch (R^2^ = 0.978), whereas the longitudinal range did not (R^2^ = 0.002). Assessment by peak residual stress alone therefore misses the interfacial gradient and the compressive zone specific to dissimilar joints.

## 1. Introduction

Dissimilar metal welded joints are increasingly used in industrial structures for both technical and economic reasons. In nuclear power plants, the main components subjected to high pressure are made of carbon steel, whereas the connecting pipework is made of stainless steel because of its superior corrosion resistance [1,2]. The rationale for such combinations is that placing each material where its particular property is required achieves an economical design without applying a high-grade steel to the entire structure.

High-manganese austenitic steel is a recent addition to this group of materials. It contains 22.5–25.5 wt % Mn and combines high strength with excellent low-temperature toughness and formability [3]. Since its inclusion in the interim guidelines of the International Maritime Organization (IMO) for cryogenic service in 2018 [4], it has been applied to liquefied natural gas (LNG) storage tanks as a substitute for 9% Ni steel [3,5]. A distinctive feature of this steel is that its coefficient of thermal expansion is lower than that of other cryogenic steels, which is advantageous with respect to the thermal stresses caused by temperature changes in cryogenic facilities [6]. In an LNG tank, the high-manganese steel is joined to the stainless steel of the loading pipework. Because this transition joint is where two steels with markedly different thermal and mechanical properties meet, its residual stress state must be examined separately.

Welding residual stress is a central concern in such joints, because tensile residual stress in a weld reduces the fatigue strength, promotes brittle fracture and accelerates stress corrosion cracking [7]. The residual stress field of a dissimilar joint is more complex than that of a homogeneous weld. Sędek et al. [8] showed that, for joints between 18G2A ferritic–pearlitic steel and 1H18N10T austenitic steel, conventional thermal stress relief is not effective, because the difference in thermal expansion of the two steels regenerates stress during cooling. Lee et al. [9] carried out a three-dimensional thermal elastic–plastic analysis of girth welds between SPPS42 carbon steel and SUS304 pipes and showed that the residual stress of a dissimilar joint is identical to that of the corresponding homogeneous joint neither in magnitude nor in distribution, and that a steep stress change is induced at the interface. Similar observations have been reported for the effect of welding conditions on the residual stress distribution in stainless-to-carbon-steel butt joints [10], for the prediction of interfacial stress in dissimilar metal pipe joints [11], and for the change in the magnitude and distribution of residual stress with heat input [12]. However, most of these studies deal with girth welds in pipes, and few have examined plate butt joints—whose restraint conditions are different—with the stress resolved into its separate components.

An attempt to isolate the individual material property factors responsible for these differences was made by Eisazadeh et al. [13]. Taking a homogeneous 304 stainless steel weld as the reference, they replaced the thermal conductivity, specific heat, yield strength and coefficient of thermal expansion of one base metal by the corresponding values of A36 carbon steel, thereby separating the role of each property, and showed that the thermal expansion coefficient is dominant. They further examined the effect of a mechanical tensioning load on residual stress formation. The authors themselves state, however, that the nonlinearity and history dependence of the elastic–plastic constitutive model kept the examination at a qualitative level, and the analysis was restricted to the longitudinal component. Moreover, because the property substitution alternated discretely between the values of the two materials, no quantitative correspondence between the magnitude of the mismatch and the magnitude of the residual stress was obtained. This study is distinguished by extending this approach to a quantitative evaluation based on a reproduction ratio, by treating the longitudinal and transverse components together, and by a parametric variation of the thermal expansion coefficient over four levels from which a relationship between the mismatch and the stress is derived. The behaviour under a tensile load is also evaluated not in terms of the magnitude of the residual stress but in terms of how much the range over which the stress rises and falls near the interface—hereafter referred to as the interfacial stress range—is relaxed by the load, in comparison with the homogeneous joint. The elastic modulus rather than the specific heat was chosen as one of the substitution factors because, for the present material combination, the specific heats of the two steels differ by only about 2%, whereas the elastic moduli differ by 13%.

The material combination examined also imposes a constraint. In austenitic–ferritic combinations, solid-state phase transformation occurs on the ferritic side during cooling, so that transformation strain and transformation plasticity are superimposed on the thermal contraction mismatch [14,15]. Recent analyses of dissimilar welds accordingly apply thermal–metallurgical–mechanical coupled models that treat the transformation effect explicitly [16]. Results obtained by substituting a single property may therefore contain the thermal expansion effect and the transformation effect without separating them. In this respect, the high-manganese steel/STS304 combination is a suitable test case. Both steels are stable face-centred cubic austenite and undergo no solid-state transformation during cooling, so that transformation strain and transformation plasticity do not intervene in the formation of residual stress. The twinning-induced plasticity exhibited by high-manganese steel is a hardening behaviour accompanying plastic deformation; it is reflected in the stress–strain relation and does not cause a volume change during cooling. In addition, the thermal expansion mismatch is not small, even though the crystal structure is the same. The coefficient of thermal expansion of the high-manganese steel used here is 9.61 × 10^−6^/°C, whereas that of STS304 is 15.96 × 10^−6^/°C, a ratio of 1.66. Judged against the coefficients reported by An et al. [6] for cryogenic steels (high-manganese steel about 9, 9% Ni steel about 12 and stainless steel about 16 × 10^−6^/°C), the present combination is among those with the largest mismatch that can be realised in cryogenic facilities. It therefore constitutes a system with a high thermal expansion contrast free from metallurgical disturbance, and is well suited to separating the contributions of the material property factors and to deriving a quantitative relationship with the mismatch.

Research on the residual stress of high-manganese steel welds has begun only recently. Lee and Seo [17,18] measured and predicted the residual stress of high-manganese steel riser pipes and derived a prediction equation from a parametric study of the radius-to-thickness ratio, but did not treat the material properties themselves as variables. The residual stress and deformation characteristics of high-manganese steel produced by friction stir welding and submerged arc welding have also been reported [19]. For dissimilar joints, An et al. [6] examined joints between cryogenic plates of high-manganese steel, 9% Ni steel and stainless steel and stainless steel pipes, and reported that the peak residual stress of each joint approaches the yield strength of the corresponding material and that, in this respect, homogeneous and dissimilar joints are not distinguishable. This study confirms this result concerning the peak value while showing that the peak value alone cannot characterise a dissimilar joint, because a steep stress gradient forms across the interface between the weld metal and the STS304 base metal, and the difference from the homogeneous joint appears not in the peak value but in this gradient and in the sign reversal of the transverse component. A steep local stress gradient may influence crack initiation, local constraint and the crack-driving force near the interface, and therefore merits consideration as a separate indicator in an integrity assessment. The behaviour when an external load is superimposed on the residual stress has been reported for homogeneous and dissimilar joints [20,21], but not for high-manganese steel dissimilar joints.

The objectives of this study are therefore fourfold. First, a three-dimensional thermal elastic–plastic finite element analysis of high-manganese steel–STS304 butt joints is performed and verified against thermocouple-measured temperature histories and residual stresses measured by the sectioning method. Because a welding thermal cycle involves temperature changes exceeding 100 °C per second, the time constant of the thermocouple directly affects both the recorded peak temperature and the shape of the cooling curve. The time constant is governed by the heat capacity of the measuring junction and by its thermal contact with the parent material, so the dynamics of the measurement are improved by reducing the wire diameter, by minimising the area over which the junction contacts the surface, and by welding the junction directly to the parent material instead of using an adhesive or a mechanical fixture. The wire diameter used here, 0.5 mm, is not that of a fine-wire installation, which is typically 25 to 100 μm; it was chosen as a compromise between response and durability, since a junction placed at the weld toe is reached by molten metal and lies within the direct influence of the arc, where a wire of that size would be likely to fail. The consequences of this choice, and the alternative junction forms available, are discussed in Section 3.1. Second, the residual stress distributions of homogeneous and dissimilar joints are compared component by component in order to characterise the interfacial stress gradient, and the effects of the welding process and of the weld metal strength are examined. Third, the yield strength, elastic modulus, thermal conductivity and coefficient of thermal expansion on the high-manganese steel side are individually replaced by the STS304 values so that the contribution of each factor is quantitatively separated, and the thermal expansion coefficient ratio is then varied parametrically over four levels to derive a quantitative relationship between the mismatch and the interfacial stress. Fourth, the redistribution of residual stress under a service-level tensile load is evaluated and its implications for integrity assessment are presented. Through these steps, the study shows that the two stress components respond in opposite ways: the longitudinal stress hardly responds to the property mismatch, whereas the transverse stress responds linearly to the thermal expansion mismatch. This contrast is supported in three ways. The extent to which each property reproduces the dissimilar-joint effect is quantified by a single indicator; a relationship linking the thermal expansion mismatch to the stress is derived; and the reason is explained by the fact that different behaviour is admissible at the interface for the two stress components. This bears directly on the question of which indicator should be used when a dissimilar joint is assessed.

## 2. Materials and Methods

### 2.1. Materials

The base metals are a cryogenic high-manganese austenitic steel and AISI 304 austenitic stainless steel (hereafter referred to as STS304, the Korean Standard designation used in this study). The high-manganese steel contains 22.5–25.5 wt % Mn and retains a stable face-centred cubic structure at room and cryogenic temperatures. The chemical compositions of the two steels are given in Table 1.

The room-temperature properties applied in the analysis are listed in Table 2. Although the two steels have the same austenitic structure, they show a clear contrast in their thermal properties. The coefficient of thermal expansion of the high-manganese steel, 9.61 × 10^−6^/°C, is 40% lower than the 15.96 × 10^−6^/°C of STS304, and its thermal conductivity of 12.2 W/m·°C is lower than the 14.6 W/m·°C of STS304 and is about one quarter of that of ordinary structural steel. The specific heats, by contrast, are 473 and 462 J/kg·°C, differing by only about 2%, and the elastic moduli are 175 and 198.5 GPa, differing by 13%. The relative magnitude of these contrasts is the basis for the selection of the substitution factors in Section 3.4.

The values in Table 2 are those actually used in the analysis and agree to within 4% with the measured values reported for the same steels (high-manganese steel 432 MPa/175 GPa, STS304 275 MPa/201 GPa) [6].

A large difference is also found in the work-hardening behaviour. At a plastic strain of 0.15, that is, at 15% deformation, the stress of the high-manganese steel is 953 MPa, 2.2 times its yield strength, whereas that of STS304 is 318 MPa, only 1.2 times its yield strength. Converting the mean slope from the yield point to a strain of 0.15 into a linear hardening rate gives 3407 MPa and 353 MPa, respectively, a difference of about one order of magnitude, which is attributed to the twinning-induced plasticity (TWIP) behaviour of the high-manganese steel [3]. The hardening rate of the weld metal, 1509 MPa, lies between those of the two base metals.

The weld metal was taken as an undermatched condition with a yield strength 20% lower than that of the base metal, and its density, elastic modulus, coefficient of thermal expansion, thermal conductivity and specific heat were set equal to those of the high-manganese steel base metal. The yield strength and the work-hardening behaviour were set differently from those of the base metal, with a stress of 580 MPa at a strain of 0.15 (hardening rate 1509 MPa). The effect of the strength mismatch of the weld metal on the residual stress is examined separately in Section 3.3.

### 2.2. Welding Specimens and Welding Conditions

**Figure 1:**
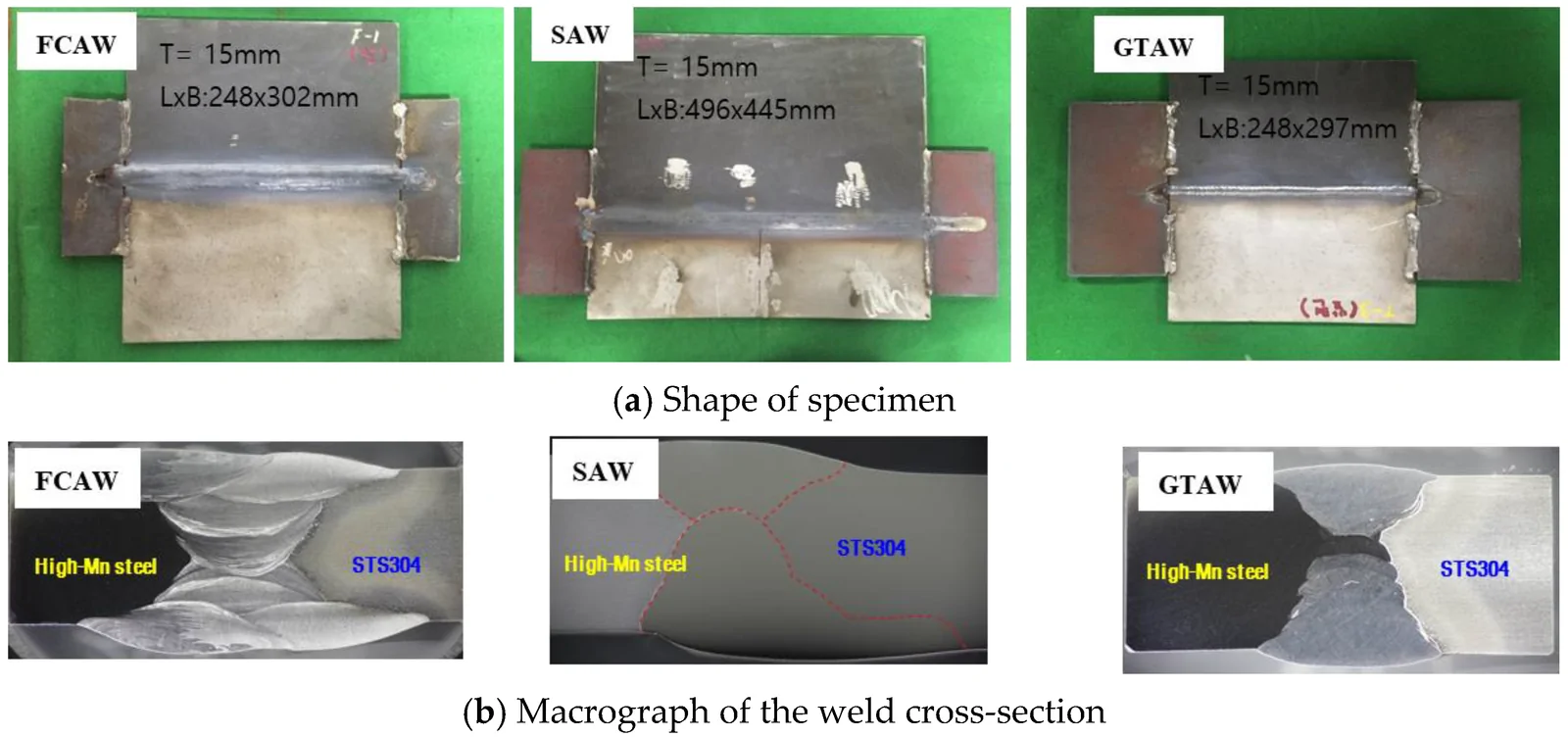

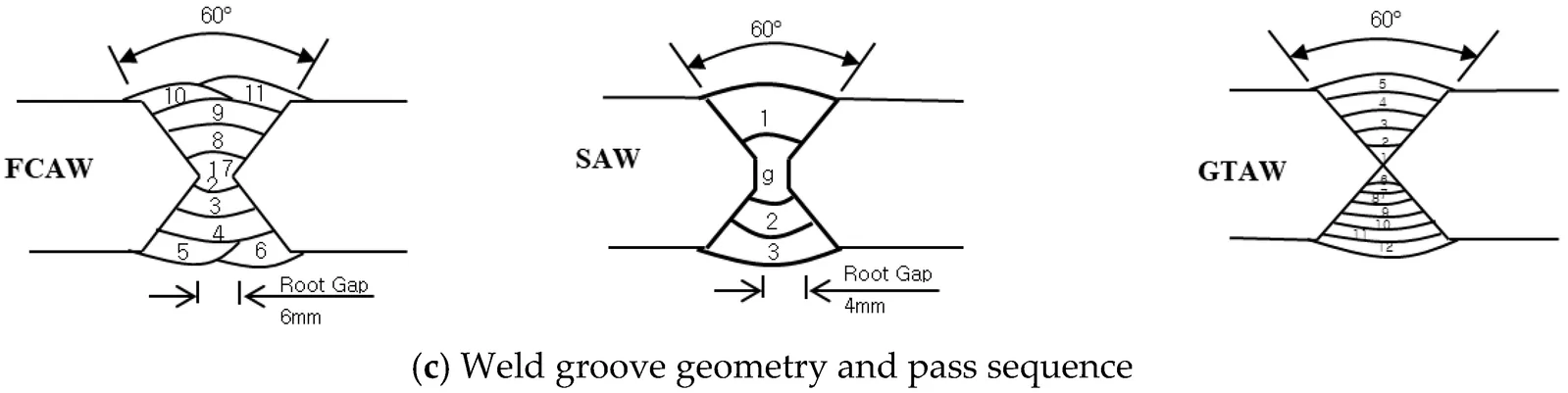
Specimen geometry, weld cross-section macrographs and groove geometry: (**a**) specimen dimensions and weld line position, (**b**) macrographs of the weld cross-section, in which the red dashed lines mark the fusion boundary where the contrast between the weld metal and the base metal is weak, (**c**) groove geometry and welding pass sequence, the numbers indicating the pass order.

The specimens are 15-mm-thick plate butt joints with the weld line at the centre of the width. Three welding processes—FCAW, SAW and GTAW—were applied, and the specimen size was varied according to the heat input of each process. The plan dimensions are 248 × 302 mm for FCAW, 496 × 445 mm for SAW, and 248 × 297 mm for GTAW. The groove angle is 60° for all three processes, and the root gap is 6 mm for FCAW and 4 mm for SAW. The specimen geometry, the macrographs of the weld cross-section and the groove geometry are shown in Figure 1, and the welding conditions are given in Table 3.

The heat input in Table 3 is the value without arc efficiency (VI/v); the effective heat input used in the analysis was obtained by multiplying it by the arc efficiency given in Section 2.5.

During fabrication, a crack occurred in the root pass of the FCAW specimen, which was re-welded after gouging, and a crack was also observed on the bead surface of the SAW specimen. These local defects and the repair history are not reflected in the analysis model.

### 2.3. Temperature-Dependent Properties

**Figure 2:**
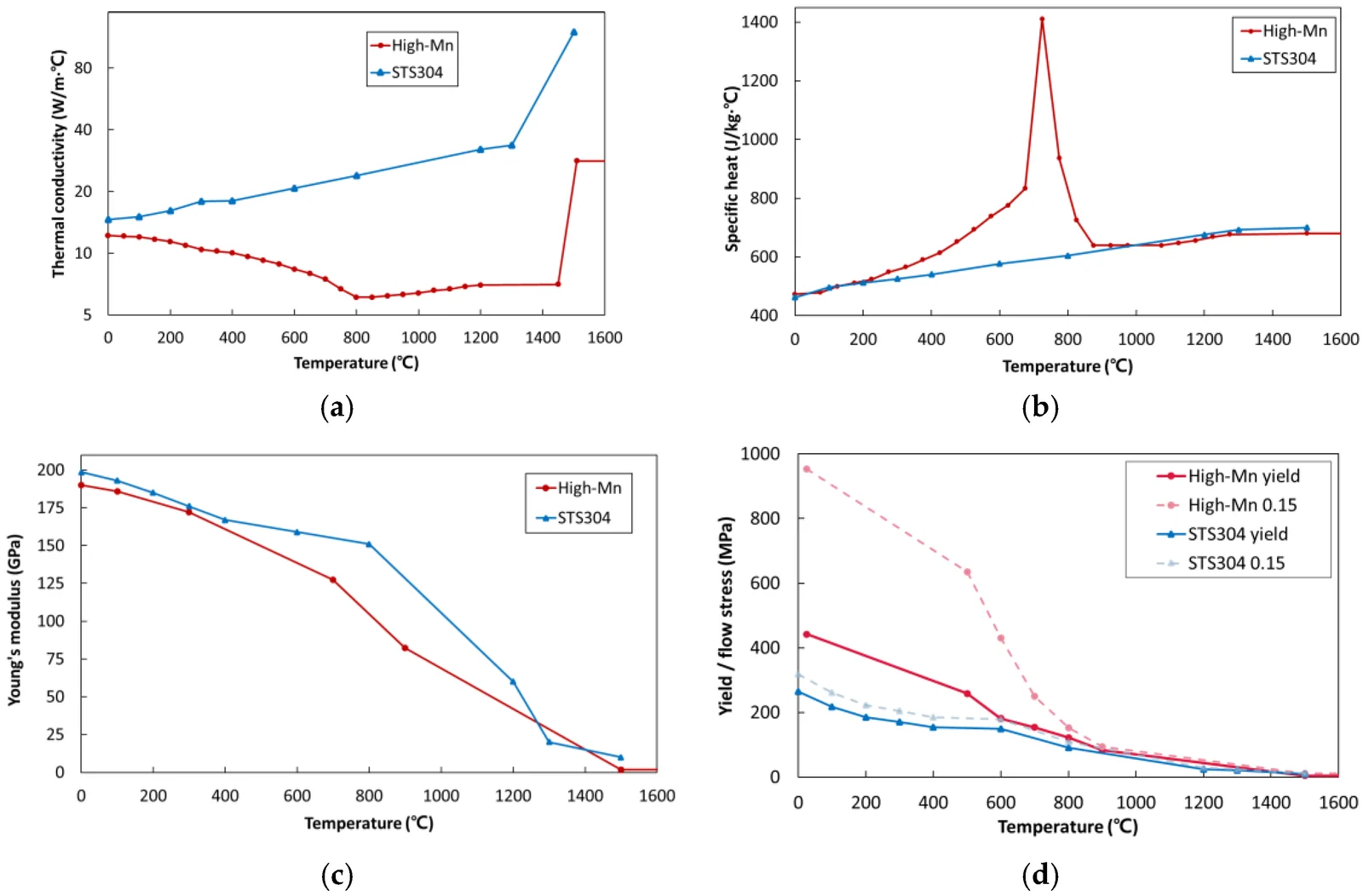
Temperature-dependent properties of the high-manganese steel and STS304: (**a**) thermal conductivity, (**b**) specific heat, (**c**) elastic modulus, (**d**) yield strength and stress at a strain of 0.15.

The temperature-dependent properties used in the analysis are shown in Figure 2. Above the melting point, a negligibly small value was assigned to the stiffness in order to simulate stress relaxation in the molten zone. The coefficient of thermal expansion was applied as a temperature-dependent property, so that the thermal strain was evaluated as *ε*_th_ = ∫*α*(T)dT. In the parametric study described in Section 3.5, the whole *α*(T) curve on the STS304 side was scaled by a constant factor, with its peak value being reduced in the prescribed proportion, so that the ratio *α*_S_/*α*_M_ took the specified levels; the values quoted in Table 2 are those at room temperature. The temperature dependence of the specific heat and elastic modulus of the high-manganese steel was taken from high-temperature property values reported for a twinning-induced plasticity steel of similar composition [6], and the properties of STS304 were taken from the literature [22]. Because both steels are stable austenite that undergoes no phase transformation, the influence of these approximations on the room-temperature residual stress is limited, although the absolute accuracy at high temperature is restricted.

### 2.4. Residual Stress Measurement by the Sectioning Method

The residual stress was measured by the sectioning method in order to verify the analysis. The method is based on measuring the strain released when the specimen is cut and back-calculating the elastic strain that was originally present.

Biaxial strain gauges were attached at the measurement positions and their initial values were recorded; the specimen was then cut in stages and the strain was measured at each stage. The released strain measured by cutting, with its sign reversed, is the elastic strain that existed at that position before cutting. Because the small piece obtained after cutting is in a state of plane stress at its surface, the residual stress is obtained from Hooke’s law as follows:

(1) $$\sigma_{L}\;=\;\frac{E}{1\;-\;\nu^{2}}\left(\varepsilon_{L}\;+\;\nu \varepsilon_{T}\right)$$

(2) $$\sigma_{T}=\frac{E}{1-\nu^{2}}\left(\varepsilon_{T}+\nu \varepsilon_{L}\right)$$

Here, *ε*_L_ and *ε*_T_ are the released strains in the direction of the weld line and in the perpendicular direction, each multiplied by (−1), and *E* and *ν* are the elastic modulus and Poisson’s ratio of the material at that position. For the dissimilar joints, the properties of the high-manganese steel, the weld metal or STS304 were applied according to the location of the measurement point.

**Figure 3:**
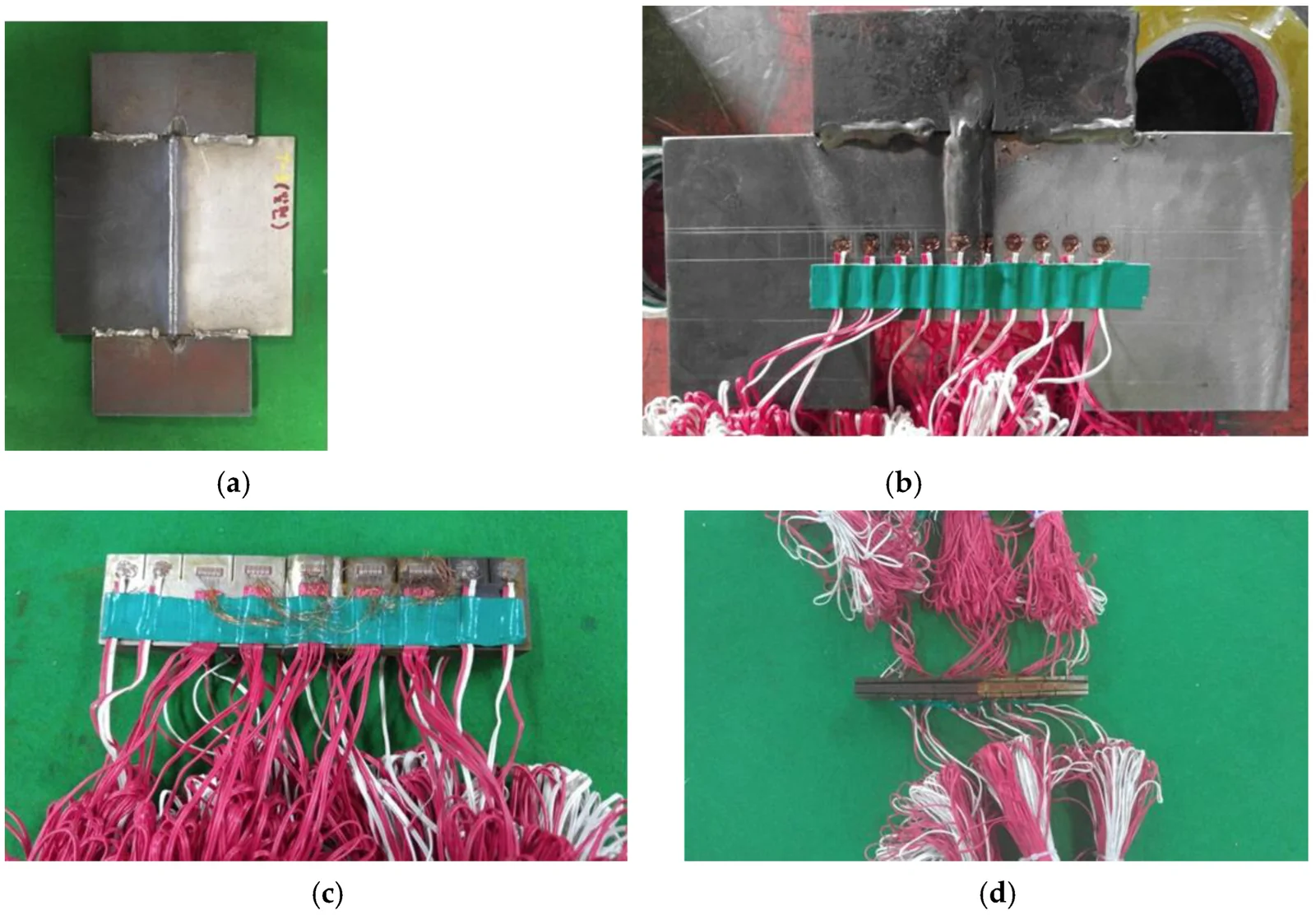
Residual stress measurement using the sectioning method: (**a**) test specimen, (**b**) strain gauge installation, (**c**) first cutting step, (**d**) second cutting step in the thickness direction.

Cutting was carried out in three stages, the sequence of which is shown in Figure 3. In the first stage, the specimen was cut to a width of 124 mm so as to include the gauged region, thereby releasing the restraint in the direction of the weld line. In the second stage, it was cut in detail into small pieces 10 mm wide in the direction perpendicular to the weld line, with 2 mm being left uncut so that the pieces were not completely separated. In the third stage, the 15 mm thickness was divided into three through-thickness sections, and the stresses of the top- and bottom-surface pieces were determined separately. The whole procedure was carried out by electrical discharge machining in order to suppress the generation of new stresses due to cutting heat.

### 2.5. Thermal Elastic–Plastic Finite Element Model

Welding is a coupled thermomechanical phenomenon, but as the influence of the mechanical field on the thermal field is negligible, a sequentially coupled approach was adopted. The temperature history was obtained by a three-dimensional transient heat conduction analysis and then applied as a thermal load in a three-dimensional nonlinear mechanical analysis. The nonlinear finite element software MSC.Marc 2024.1 was used.

In the mechanical analysis, a rate-independent elastic–plastic constitutive equation with the von Mises yield criterion and the isotropic hardening rule was used, and the temperature-dependent yield strength, elastic modulus and coefficient of thermal expansion were taken into account. Because both high-manganese steel and STS304 retain a stable austenitic structure down to room temperature and undergo no solid-state transformation during cooling, transformation strain and transformation plasticity were not considered. The effect of the deformation-induced twinning of the high-manganese steel is included in the stress–strain relation at each temperature. The sequential deposition of the weld metal was simulated by the element activation/deactivation technique, and the nonlinear solution was obtained by the full Newton–Raphson iteration method.

The welding heat source was modelled by Goldak’s double-ellipsoid model [23]. The heat flux distributions inside the front and rear semi-ellipsoids are as follows:

(3) $$q_{f}\left(x,\;y,\;z\right)\;=\;\frac{6\sqrt{3}f_{f}Q}{abc_{f}\pi \sqrt{\pi }}\;exp\left(-\frac{3x^{2}}{a^{2}}\;-\;\frac{3y^{2}}{b^{2}}\;-\;\frac{3z^{2}}{{c_{f}}^{2}}\right)$$

(4) $$q_{r}\left(x,\;y,\;z\right)=\frac{6\sqrt{3}f_{r}Q}{abc_{r}\pi \sqrt{\pi }}\;exp\left(-\frac{3x^{2}}{a^{2}}-\frac{3y^{2}}{b^{2}}-\frac{3z^{2}}{{c_{r}}^{2}}\right)$$

Here, *Q* = *η*VI is the effective heat input, *η* is the arc efficiency, *V* is the arc voltage and *I* is the arc current; *a* is the half-width of the molten pool, *b* is its depth, and *c*_f_ and *c*_r_ are the front and rear semi-axis lengths, while *f*_f_ and *f*_r_ are the front and rear heat-distribution coefficients satisfying *f*_f_ + *f*_r_ = 2. The heat source shape parameters *a*, *b*, *c*_f_ and *c*_r_ were adjusted individually for each process so that the fusion boundary and the heat-affected zone width observed in the macrographs agreed with the analysis. Arc efficiencies of 0.8 for FCAW, 0.9 for SAW and 0.6 for GTAW were applied. These follow the calorimetric values of DuPont and Marder [24] (0.84 for consumable electrode processes and 0.67 for GTAW) and the distribution of reported GTAW values compiled by Stenbacka [25]; SAW, in which the flux covering suppresses radiation losses, was set highest, and GTAW, in which part of the arc heat is lost to the tungsten electrode and the shielding gas, was set lowest. The heat-distribution coefficients *f*_f_ = 0.6 and *f*_r_ = 1.4 proposed by Goldak et al. [23] were used so that *f*_f_ + *f*_r_ = 2. The resulting effective heat inputs for the filling passes are 12.0 kJ/cm for FCAW, 31.1 kJ/cm for SAW and 11.9 kJ/cm for GTAW.

Heat loss by convection and radiation at the specimen surface was applied as a temperature-dependent combined heat-transfer coefficient with values of 4 W/m^2^·°C at room temperature and 13.6 W/m^2^·°C at 1500 °C [22]. The thermal conductivity above the melting point was increased about fourfold in order to approximate the enhancement of heat transfer by convection inside the molten pool.

**Figure 4:**
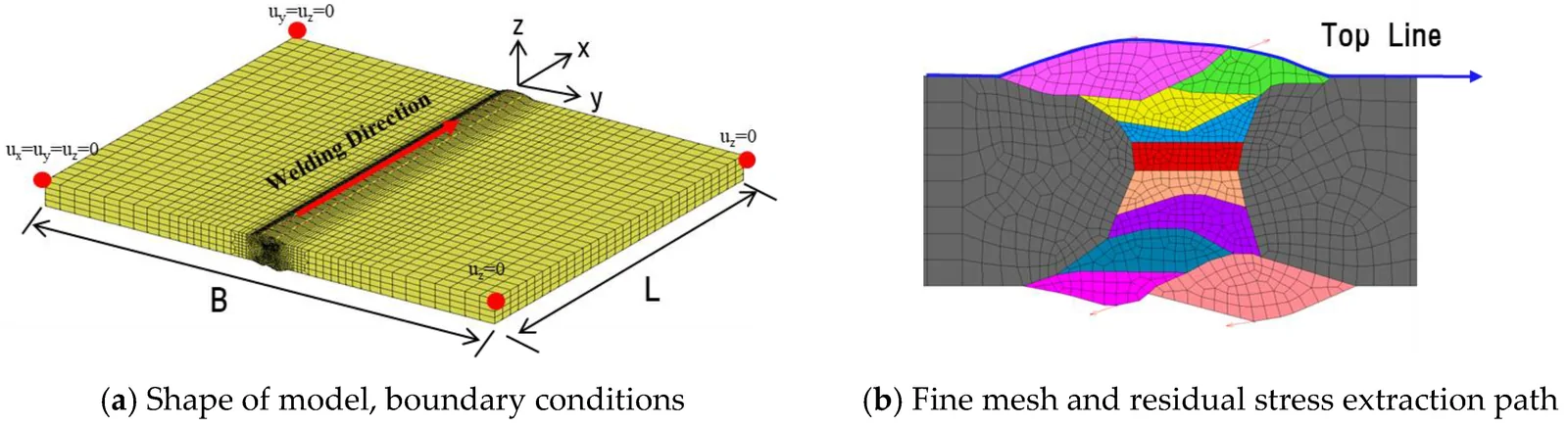
Finite element model: (**a**) model geometry and boundary conditions, (**b**) mesh in the vicinity of the weld and the residual stress extraction path.

The finite element model and the boundary conditions are shown in Figure 4. Eight-node hexahedral elements were used. The weld and its vicinity were finely divided in order to capture the steep temperature gradient of the moving heat source and the high stress gradient at the interface, and the element size was increased with distance from the weld line. The smallest element size is 0.5 mm, and the model has 35,464 elements and 39,072 nodes. The adequacy of the mesh was confirmed by a preliminary mesh sensitivity study. The thermal and mechanical analyses used the same mesh, differing only in element type, so that the nodal data could be mapped. The mechanical boundary conditions were applied so as to constrain only the rigid body motion of the specimen.

The residual stress was extracted along a path perpendicular to the weld line at the mid-length cross-section, where the effects of the welding start and stop are excluded, at two positions on the top and bottom surfaces.

### 2.6. Analysis Cases

The analysis cases carried out in this study are summarised in Table 4.

The property substitution of group C was carried out by replacing only one specified property of one base metal by the STS304 value, taking the homogeneous joint as the reference, while the properties of the other base metal and of the weld metal were fixed at the high-manganese steel values. This single-factor substitution makes it possible to separate the individual contribution of each property mismatch.

The substitution factors were selected as the four thermal and mechanical properties that are directly involved in residual stress formation and that differ most between the two steels. The specific heat was excluded because the difference between the two steels is only about 2% (Table 2), and the elastic modulus, which differs by 13%, was included instead. The density was also excluded because it differs by only 3% and contributes to the thermal analysis only through the heat capacity, together with the specific heat.

In group D, only the coefficient of thermal expansion on the STS304 side was varied over four levels by scaling its temperature-dependent curve, with the value of 1.66 corresponding to the actual ratio of the two steels. The applied stress of 212 MPa in group E corresponds to 80% of the yield strength of the STS304 base metal. The analyses of groups B and E were carried out with models separate from those of groups A and C. In Section 3.3 and Section 3.7, the results are therefore discussed on the basis of the relative change within each series.

## 3. Results and Discussion

### 3.1. Verification of the Analysis Method

**Figure 5:**
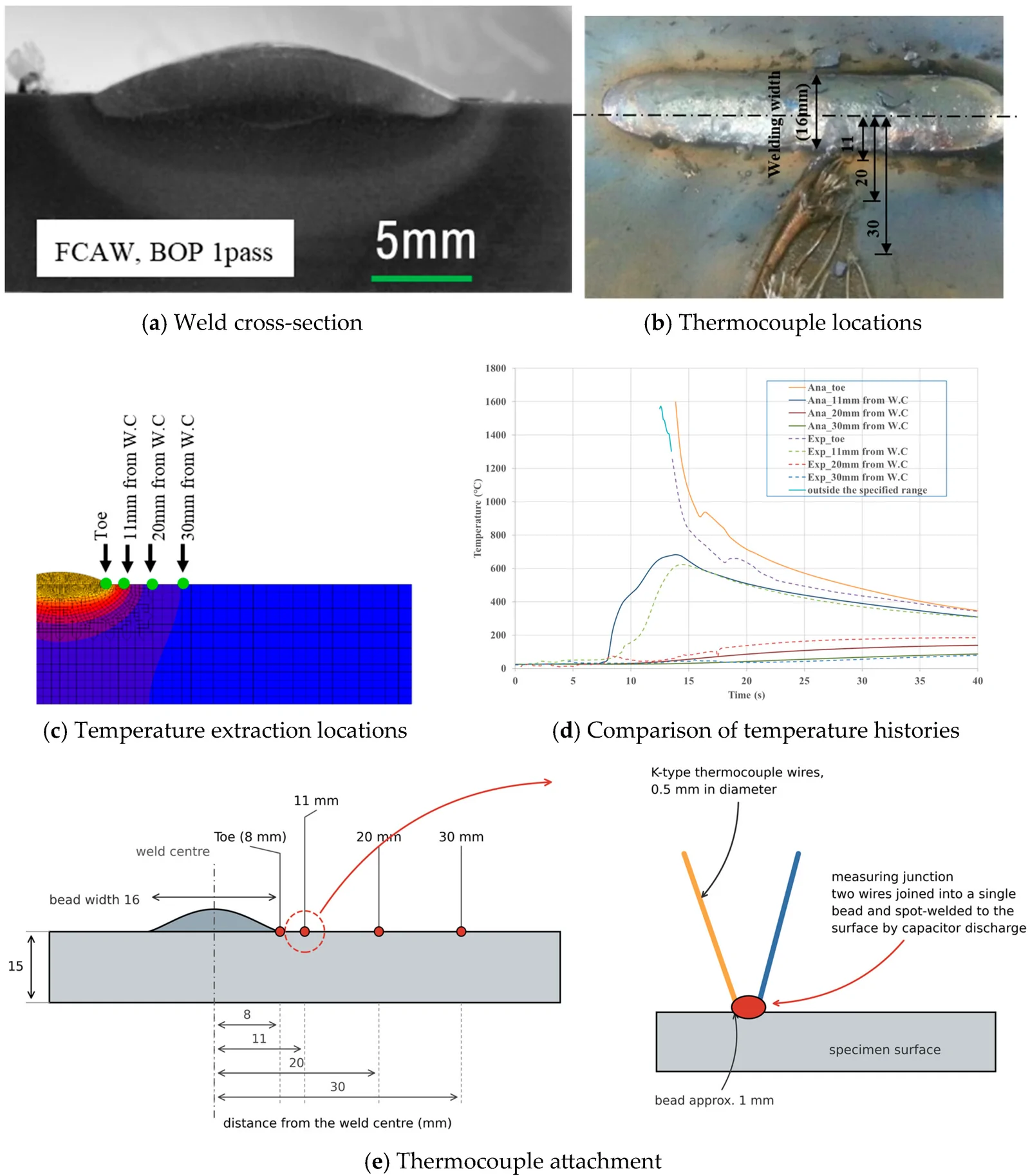
Temperature measurement conditions and analysis results: (**a**) weld cross-section, (**b**) thermocouple locations (8, 11, 20 and 30 mm from the weld centre; the bead width is 16 mm, so the position at 8 mm is the weld toe), (**c**) temperature extraction locations on the cross-section of the analysis model, (**d**) comparison of measured and calculated temperature histories, in which the portion of the measurement above the specified range of the thermocouple is plotted as a separate series, (**e**) schematic of the thermocouple attachment; the junction detail is enlarged.

Because the reliability of the mechanical analysis depends on the accuracy of the temperature field, the thermal analysis was verified first. During the root pass of FCAW, four K-type (chromel–alumel) thermocouples with a wire diameter of 0.5 mm, with a specified range of −200 to +1260 °C, were attached at 8, 11, 20 and 30 mm from the weld centre. The bead width measured from the macrograph is 16 mm, so the position at 8 mm coincides with the weld toe. For each thermocouple, the two wires were first joined into a single bead approximately 1 mm in diameter, and that bead was then spot-welded directly to the specimen surface by capacitor discharge, in which the charge stored in a capacitor is released as a high-current pulse of millisecond duration that fuses the bead to the surface. Because the discharge is so short, the heat input is confined to the immediate vicinity of the junction, and a metallurgical bond is obtained without an adhesive or a mechanical fixture. The attachment is shown schematically in Figure 5e. It should be noted that a junction of this form is a volumetric one and is not the fastest arrangement available. In an intrinsic junction, the two wires are welded separately to the surface a short distance apart so that the parent material itself completes the circuit; the thermal mass of the junction is thereby largely removed and the response is faster than that of a welded bead [26,27]. The bead form was used here for its durability in the immediate vicinity of the weld. The diameter quoted above is a nominal value derived from the wire diameter; the junction was not measured. The temperature histories were recorded at intervals of 0.1 s and compared with the analysis values at the same positions. The results are shown in Figure 5 and Table 5. Comparing measurement and analysis in this way is a procedure widely used for verifying the temperature field of thin-plate welds [28].

The window of 40.7 s refers only to the thermal validation analysis of the FCAW root pass, for which the thermocouple records are available, and not to the complete multi-pass analysis; the full model was carried through all passes and cooled to room temperature before the residual stress was extracted. Because residual stress is determined during the cooling period in which the material recovers its strength, the cooling behaviour rather than the peak temperature is what matters in the verification. At 40.7 s, the end of the analysis, the temperature at the toe was 338.1 °C measured against 339.7 °C calculated, while that at the position 11 mm from the weld centre was 303.7 °C against 303.5 °C, that is, virtually identical. The 800 → 500 °C cooling time at the toe was 9.1 s measured against 10.3 s calculated, a difference of 13.2%; the corresponding 600 → 400 °C cooling time at 11 mm from the weld centre was 11.2 s against 12.9 s, a difference of 15.8%. Since the residual stress is governed by the cooling period, over which the material recovers its strength, differences of this order are regarded as acceptable. At the toe, the largest value recorded was 1573.3 °C. This lies above the specified upper limit of 1260 °C for a K-type thermocouple, beyond which the relation between the electromotive force and the temperature is no longer guaranteed; it is therefore an indication rather than a valid measurement and was not used in the verification. The values exceeding that limit are shown separately in Figure 5d and are identified as such in Table 5. This does not alter the verification, because the comparison with the analysis begins at 13.86 s, when the weld metal element is activated, and by that instant the measured temperature has already fallen to 1125 °C, within the range of the instrument. In Figure 5d, the record within the specified range begins at 13.6 s and the calculated curve begins at 13.86 s. Neither is a gap in the record: the portion of the measurement above the specified range is plotted as a separate series, and the calculated curve exists only after the weld metal element at that position has been activated, with no temperature being defined there beforehand. The calculated value of 1600.0 °C at the toe is likewise not a prediction but the activation temperature assigned to the weld metal elements; what is verified at the toe is the cooling curve after element activation, for which the RMSE is 121.5 °C. At 20 and 30 mm from the weld centre, the measured peaks occur at 43.7 s and 86.4 s, after the end of the analysis, so the peak values cannot be compared; at the end of the analysis, the calculated value at 20 mm is 24.4% lower. However, the peak temperatures at these two positions, 185.7 °C and 108.5 °C, lie in a range in which the base metals do not deform plastically (Figure 2), so the influence on the residual stress prediction is limited. The thermal analysis is therefore judged to reproduce, at a reliable level, the cooling behaviour near the weld that governs residual stress formation. The shape and size of the measuring junctions were not recorded at the time of the test, which is a limitation of this measurement.

**Figure 6:**
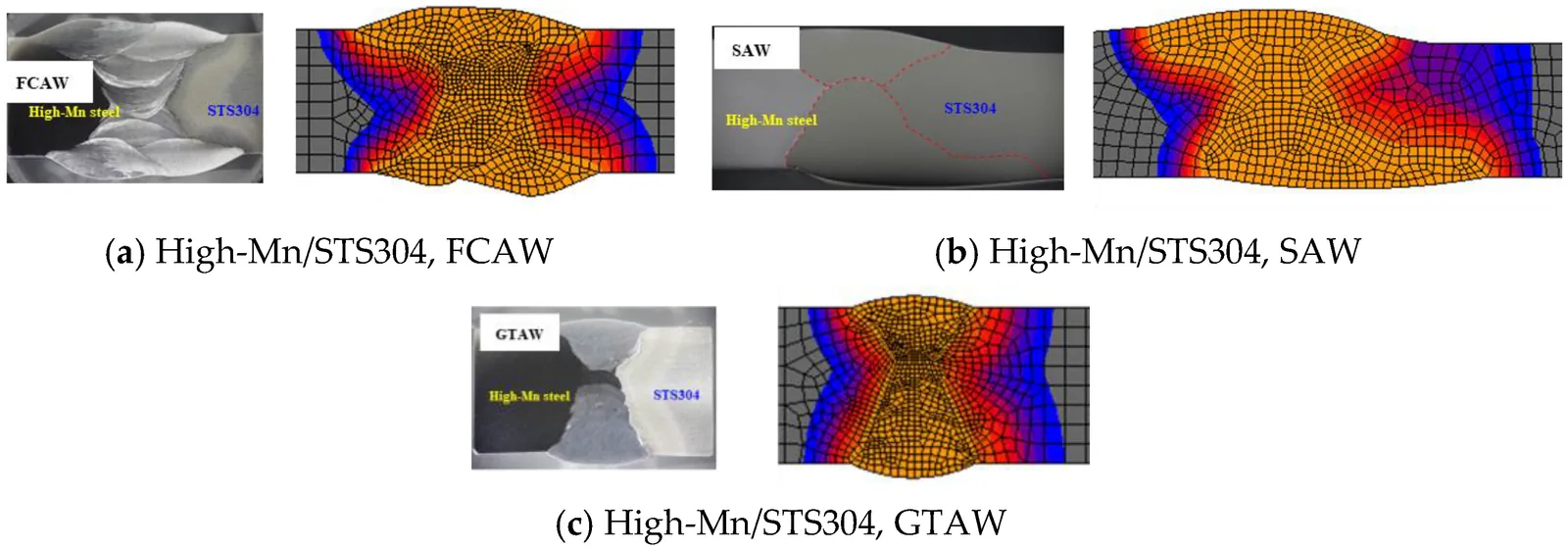
Comparison of the cross-section macrographs of the dissimilar joints (high-manganese steel/STS304) with the calculated heat-affected zone shape (700 °C isotherm): (**a**) FCAW, (**b**) SAW, (**c**) GTAW. In each pair, the macrograph is on the left and the calculated temperature field on the right, the colours being temperature contours. The red dashed lines in the SAW macrograph mark the fusion boundary, traced because the contrast is weak there.

The shapes of the fusion boundary and the heat-affected zone were also compared with the macrographs (Figure 6). Taking the 700 °C isotherm as the boundary of the heat-affected zone, the calculated width was consistent with that of the macrographs for all three processes. The heat-affected zone on the high-manganese steel side is about 1.5 times wider than in ordinary structural steel, because the thermal conductivity of the high-manganese steel, 12.2 W/m·°C, is only about one quarter of that of ordinary structural steel, so that heat accumulates locally near the weld. This shows that the thermal analysis properly reflects the difference in thermal properties between the two steels.

**Figure 7:**
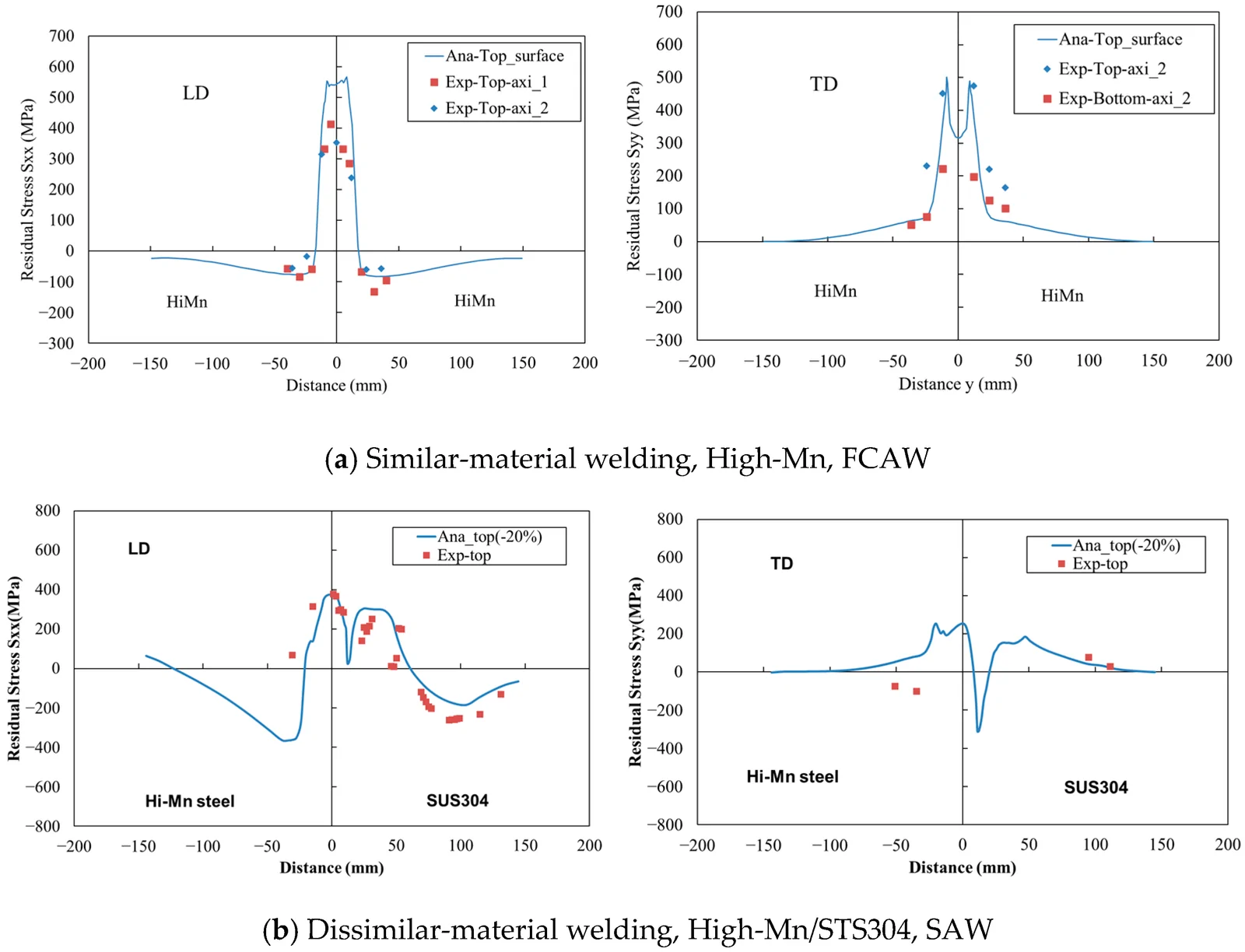

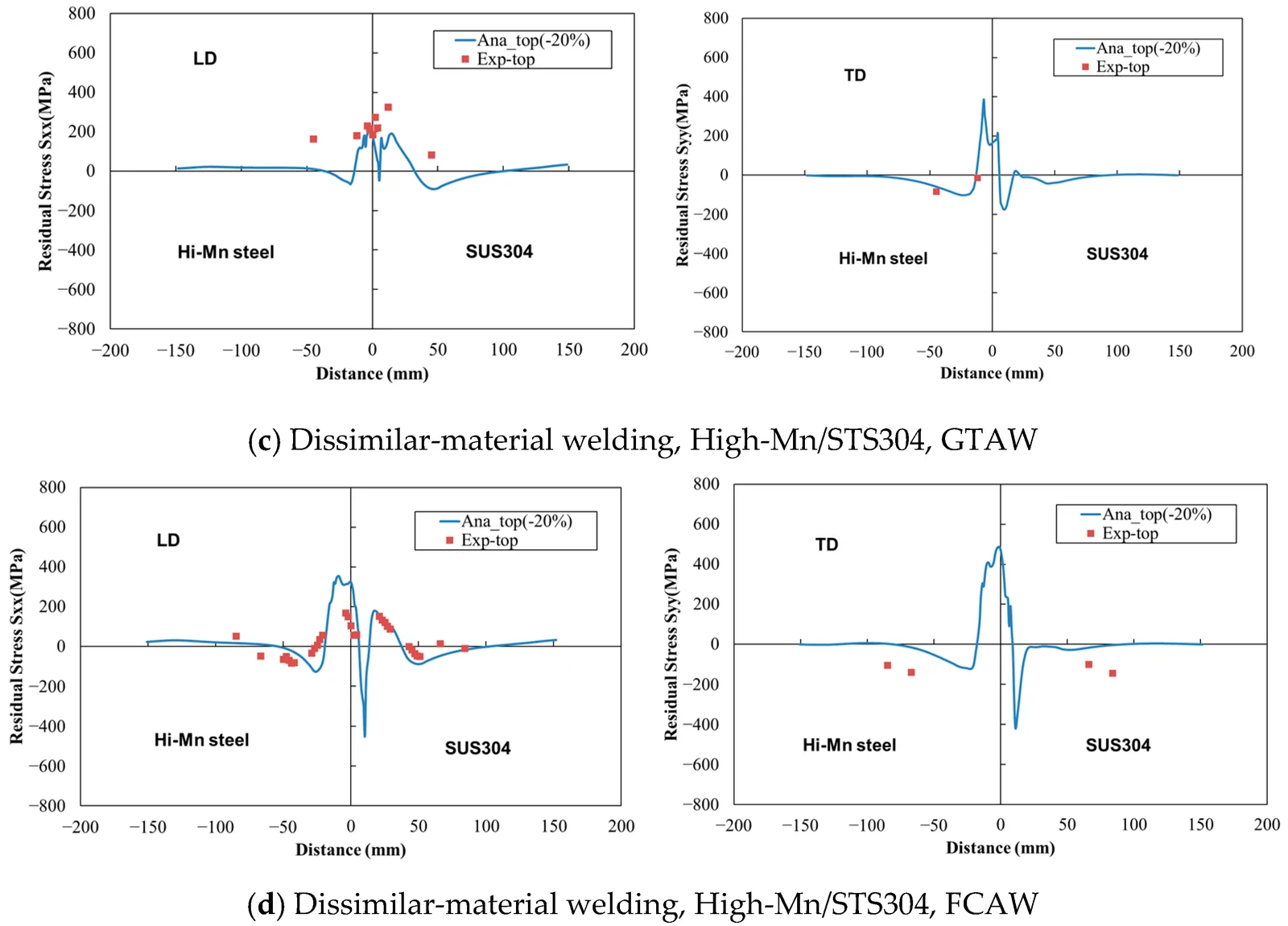
Comparison of measured and calculated residual stresses (in each panel the longitudinal stress *σ*_L_ is on the left and the transverse stress *σ*_T_ on the right): (**a**) homogeneous high-manganese steel joint, FCAW, (**b**) dissimilar high-manganese steel/STS304 joint, SAW, (**c**) dissimilar joint, GTAW, (**d**) dissimilar joint, FCAW.

The residual stresses measured by the sectioning method were then compared with the analysis (Figure 7). Measurements were made on two series, the homogeneous joint (high-manganese steel/high-manganese steel, FCAW) and the dissimilar joints (high-manganese steel/STS304, FCAW, SAW and GTAW), and in each series the longitudinal (*σ*_L_) and transverse (*σ*_T_) components were taken on the top and bottom surfaces. The calculated values were matched by linear interpolation at the coordinates of each measurement point. The results are summarised in Table 6.

It should be made clear that the agreement between the calculation and the measurement is not at the same level as for the temperature field. The RMSE values of the two series, 103.3 MPa and 127.0 MPa, correspond to 16–22% of the peak residual stress of the respective joints (657.2 MPa and 580.2 MPa), and the maximum deviation reaches 438.5 MPa in the dissimilar series. The analysis therefore does not reproduce the value at every individual measurement point within the measurement scatter, and the agreement should be regarded as a correspondence of trend, that is, of the overall shape of the distribution and of the characteristic locations.

Several factors act together to produce these deviations. Because the sectioning method back-calculates stress from the strain released by cutting, the cutting sequence, the size of the pieces and the accuracy of gauge attachment are directly reflected in the result, and the plane stress assumption is itself only an approximation for a 15 mm thick plate, so the measurement uncertainty is large. In addition, the stress gradient near the interface exceeds 200 MPa/mm (Section 3.2), so a positional error of only 1 mm produces a difference of more than 200 MPa. Furthermore, the number of measurement points varies from 2 to 29 depending on the combination, so the statistics of an individual combination are weakly representative.

The spatial distribution of the deviations indicates which of these factors dominates. The deviations are not confined to the interface: averaged over the near-weld region (|x| < 60 mm), they are 136.0 MPa on the high-manganese steel side against 88.4 MPa on the STS304 side, and the mean signed deviation is −39.0 MPa against +14.1 MPa, so the analysis underestimates on one side and overestimates on the other. Beyond 60 mm, the mean deviation falls to 55.2 MPa. The two largest deviations, 438.5 MPa and 430.4 MPa, occur at x = −20 mm and x = −31 mm, that is, in the high-manganese steel base metal rather than at the interface at x ≈ +8.5 mm. This asymmetry is consistent with the temperature-dependent properties of the high-manganese steel having been taken from a steel of similar composition, whereas those of STS304 are established literature values (Section 2.3). Near the interface itself, a further contribution arises from the gauge length: applying a 10 mm moving average to the calculated profile, corresponding to the width of the pieces released by sectioning, changes the profile by up to 322 MPa for *σ*_L_ and 257 MPa for *σ*_T_, with the largest change coinciding with the interface. A local extremum developed over 1 to 2 mm therefore cannot be resolved by this measurement. This effect alone does not account for the entire discrepancy, however, since applying the same average reduces the RMSE only from 128.9 to 126.0 MPa. Electrical discharge machining introduces little cutting force or heat and is expected to contribute little, although it cannot be excluded entirely.

Nevertheless, it is significant that the RMSE values of the two series (103.3 MPa against 127.0 MPa) and the mean deviations (+19.0 MPa against +7.3 MPa) are comparable. Because the analysis does not become systematically worse for the dissimilar joint than for the homogeneous joint, no bias associated with dissimilarity has been introduced. Moreover, all the examinations from Section 3.2 onwards are relative comparisons between analyses carried out under the same modelling assumptions, so the systematic errors arising from the factors above may partly cancel in the comparison. Accordingly, the analysis results are used in this study not to predict absolute values of residual stress but to discriminate relative differences between joints and between property conditions, and the absolute magnitudes presented should be read as values carrying an uncertainty of the order of 100 MPa.

### 3.2. Residual Stress Gradient and Compressive Zone at the Interface of the Dissimilar Joint

**Figure 8:**
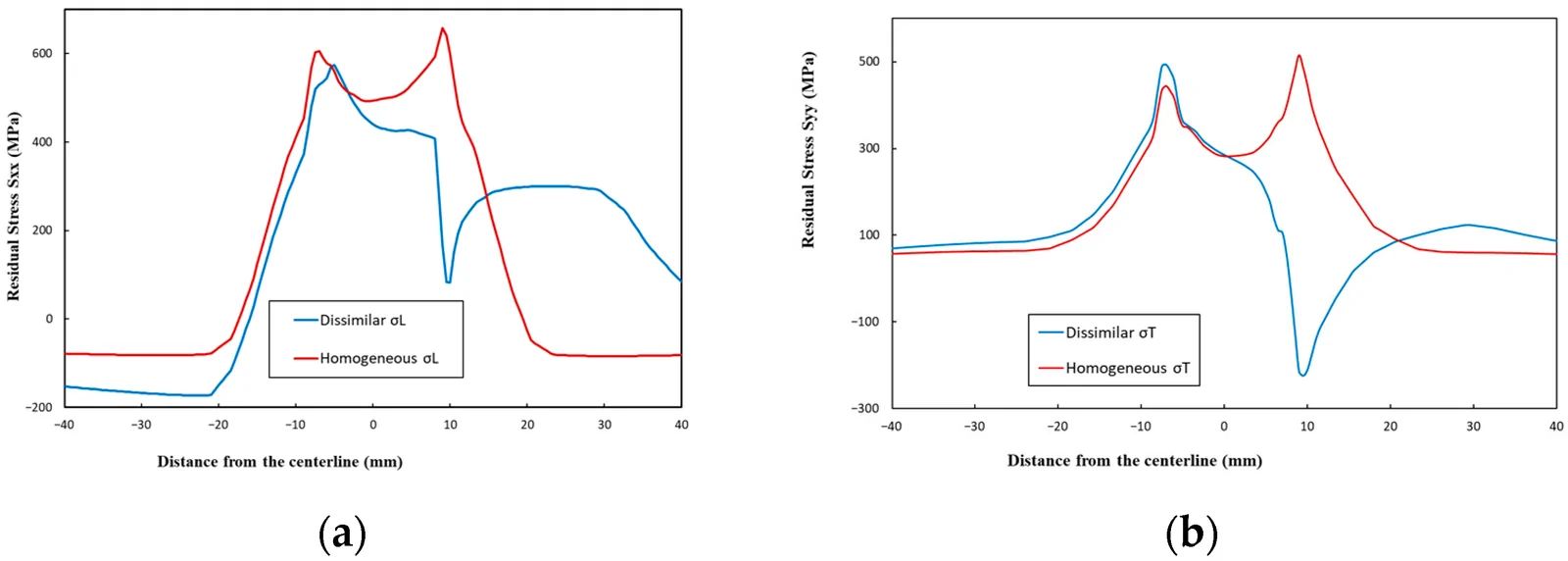
Residual stress distributions of the homogeneous and dissimilar joints (top surface): (**a**) longitudinal stress *σ*_L_, (**b**) transverse stress *σ*_T_.

As confirmed in Section 3.1, the analysis reproduces the two joints with comparable accuracy; therefore, in what follows, the difference between the homogeneous and dissimilar joints is treated as a relative comparison. The residual stress distributions of the homogeneous and dissimilar joints are compared component by component in Figure 8, and the principal values are summarised in Table 7.

The behaviours of the two components contrast sharply. The longitudinal stress *σ*_L_ remains at a similar level in both joints. The maximum is 657.2 MPa for the homogeneous joint and 580.2 MPa for the dissimilar joint, somewhat lower for the latter, but the stress ranges are 741.2 MPa and 753.0 MPa, respectively, differing by only 1.6%. Both joints show a self-equilibrating distribution in which the maximum tensile stress occurs in the weld and changes to compression towards the base metal.

The difference of 77.0 MPa in the maximum can hardly be regarded as significant either. It is smaller than the deviation between the calculation and the measurement shown in Table 6 (103.3 MPa for the homogeneous and 127.0 MPa for the dissimilar series). Therefore, distinguishing the two joints on the basis of the peak value is not justified even from the standpoint of measurement precision.

The transverse stress *σ*_T_, by contrast, is entirely different. In the homogeneous joint, tensile stress is distributed over the entire range and the minimum is +39.2 MPa, so no compression appears. In the dissimilar joint, however, the analysis predicts that the stress decreases sharply near the weld metal–STS304 interface 8.5 mm from the weld centre and reaches a compressive stress of −228.4 MPa. It should be emphasised that this local extremum is a computed value: the sectioning method releases strain over pieces about 10 mm wide, so it cannot resolve a feature that develops over about 1 mm, and the measured points in Figure 7 therefore neither confirm nor contradict the predicted peak directly. The stress range increases by 51.6%, from 475.9 MPa to 721.6 MPa.

The location of the maximum stress gradient also differs. In the homogeneous joint, the maximum gradient of *σ*_L_ occurs at x = +10.0 mm and that of *σ*_T_ at x = −8.0 mm, symmetrically about the weld centre. In the dissimilar joint, both components show their maximum gradient at x = +8.5 mm, which coincides with the interface between the weld metal and the STS304 base metal, that is, with the groove boundary. The magnitude of the gradient increases from 120.1 to 265.4 MPa/mm for *σ*_L_ and from 134.5 to 195.1 MPa/mm for *σ*_T_. The fact that the maximum gradients of the two components coincide at the material boundary indicates that this gradient originates from the material discontinuity rather than from the welding thermal history.

These results show that the characteristic feature of a dissimilar joint lies not in the magnitude of the peak stress but in the steep stress gradient and the local compressive zone at a particular location. If the peak residual stress alone is used as the indicator, the difference between the two joints cannot be identified; this point is discussed again in Section 3.6.

### 3.3. Effects of the Welding Process and of the Weld Metal Strength

Whether the steep stress gradient at the interface originates from the welding process or from the strength mismatch of the weld metal was examined. The analyses in this section were carried out with a model separate from that of Section 3.2, so the absolute values are not compared directly with Section 3.2 and the relative change within each series is examined instead.

For the three welding processes (FCAW, SAW and GTAW), the absolute magnitude of the stress varied with the heat input, but the steep stress change at the interface was common only to the dissimilar joints and was not observed in the homogeneous joint for any process. The results obtained by varying the yield strength of the weld metal over four levels from 353.6 MPa to 530.4 MPa are given in Table 8.

When the yield strength of the weld metal increased by 50%, from 353.6 MPa to 530.4 MPa, the maximum stress rose by 33.0%, from 571.1 MPa to 759.6 MPa. This is expected, since the magnitude of the stress in the weld is governed by the plastic resistance of the weld metal. Under the same conditions, however, the minimum stress changed only by 18.0 MPa, from 49.0 MPa to 67.0 MPa, and no transition to compressive stress occurred at any level.

The strength of the weld metal therefore governs the magnitude of the stress in the weld but is not involved in the interfacial stress gradient and compressive zone that are peculiar to the dissimilar joint. The interfacial feature was likewise observed consistently in all the process cases analysed. The stress gradient at the interface is thus judged to originate not from the process conditions but from the difference in properties between the two base metals.

### 3.4. Separation of the Contribution of Each Material Property

**Figure 9:**
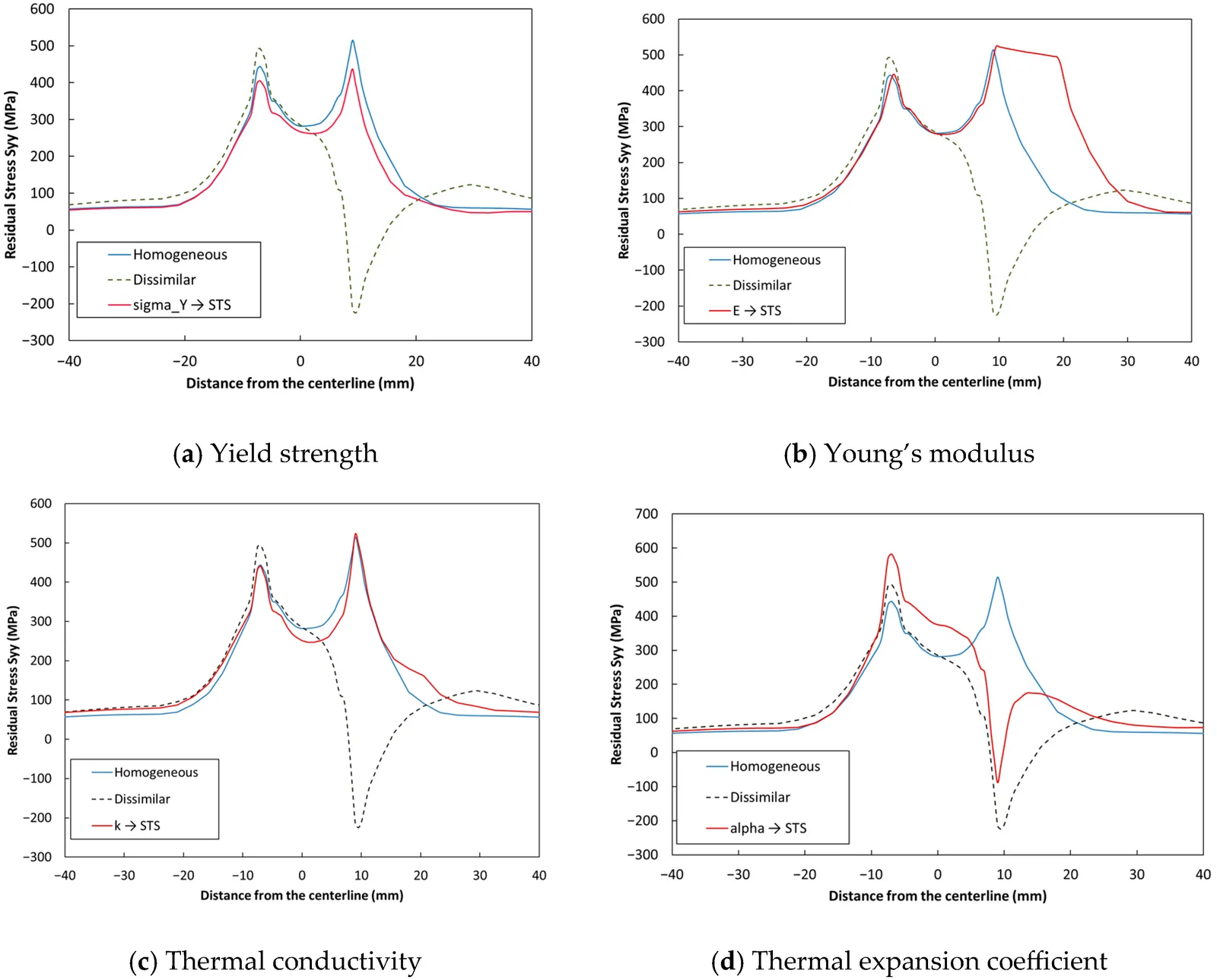
Effect of single-property substitution on the transverse residual stress (top surface): substitution of (**a**) the yield strength, (**b**) the elastic modulus, (**c**) the thermal conductivity and (**d**) the coefficient of thermal expansion by the STS304 values.

Since the process and the weld metal strength were excluded in Section 3.3, the difference in properties between the two base metals remains as the principal candidate. Four properties were examined; the work-hardening curve, Poisson’s ratio, and the heat capacity were not substituted. Therefore, the ranking below applies to the four properties examined and not to every property difference between the two steels. Figure 9 shows the results obtained by replacing the properties of one base metal, one at a time, with the STS304 values, taking the homogeneous joint as the reference. In the figure, the blue solid line is the homogeneous joint, the black dashed line is the dissimilar joint, and the red solid line is each substitution case. When the yield strength, elastic modulus or thermal conductivity is substituted, the red solid line follows the blue solid line almost exactly, whereas only the substitution of the coefficient of thermal expansion reproduces the behaviour of the black dashed line.

Two stress changes were defined in order to quantify the contribution of each factor. One is the total change caused by dissimilarity, *Δ*_full_ = *σ*_Dissi_ − *σ*_Homo_, and the other is the change when only one property is substituted, *Δ*_C_ = *σ*_C_ − *σ*_Homo_. Here, *σ*_Homo_ and *σ*_Dissi_ are the residual stresses of the homogeneous and dissimilar joints, and *σ*_C_ is the residual stress obtained from the analysis in which only property C on the high-manganese steel side has been changed to the STS304 value, with C representing the yield strength, elastic modulus, thermal conductivity, or coefficient of thermal expansion. From these, a projection coefficient R, referred to below as the reproduction ratio, was evaluated as follows.

(5) $$R=\frac{\left\langle \Delta_{C},\;\Delta_{full}\right\rangle }{\left\langle \Delta_{full},\;\Delta_{full}\right\rangle }\times 100\;(\%)$$

Here, ⟨ , ⟩ denotes the inner product over the whole stress path. *R* is the projection of *Δ*_C_ onto *Δ*_full_, so it measures how much of the dissimilarity effect is recovered along that direction and does not depend on the value at any particular point. Two consequences should be noted. *R* = 100% does not imply that the two distributions coincide, because the component of *Δ*_C_ orthogonal to *Δ*_full_ is not penalised, and *R* may exceed 100% when the projection overshoots. *R* is therefore used here as *a* directional measure of contribution and not as *a* goodness-of-fit index.

When interpreting the contribution of each factor, the consistency of the sign must be considered as well as the magnitude. The elastic modulus gives 47.2% for *σ*_L_ on the top surface, the largest of the four factors, but −22.7% for *σ*_T_ on the top surface, with the opposite sign. Substituting the elastic modulus therefore changes the stress in the direction of dissimilarity for one component and in the opposite direction for another, so it cannot be regarded as the governing factor. The results are given in Table 9.

The thermal conductivity gives 27.4% only for *σ*_T_ on the bottom surface and less than 2% for the other three indicators. The yield strength contributes consistently positively in the range of 10.8–21.3%, but the magnitude is small. The coefficient of thermal expansion, by contrast, contributes consistently positively for all four indicators, in the range of 30.6–98.1%, so that its mean of 57.9% greatly exceeds the sum of the other three factors (30.7%). It should be noted that for a factor such as the elastic modulus, whose sign is opposite for different components, the mean value itself has no physical meaning, so the means in Table 9 should be interpreted only after the consistency of the sign has been checked.

The occurrence of the interfacial compressive zone is even clearer (Table 10). No compressive stress occurs at all in the homogeneous joint, whereas a compression of −228.4 MPa appears in the dissimilar joint. Of the four factors, only the coefficient of thermal expansion reproduces this sign reversal; after substitution of the other three, the minimum remains in the range of +36 to +46 MPa.

When all four properties are substituted simultaneously, the reproduction ratio is 102.4%, showing that the residual stress behaviour of the dissimilar joint is explained by the property differences of the two base metals alone. The difference between the sum of the individual contributions (88.6%) and the result of simultaneous substitution (102.4%) is only 13.8 percentage points. The reproduction ratio of 118.0% for *σ*_T_ on the bottom surface exceeds 100% because, upon simultaneous substitution, the interaction between the factors acts in the same direction as dissimilarity and slightly exceeds the sum of the individual contributions. The discrepancy of 13.8 percentage points is reported here as a quantitative value rather than being characterised as small, since a projection-based comparison evaluates only the component along *Δ*_full_ and cannot by itself bound the interaction; a direct vector measure such as ‖*Δ*all − Σ*Δ*i‖/‖*Δ*_full_‖ would be required for that purpose. Within this limitation, the contributions along the dissimilarity direction are close to additive, which supports the present approach of substituting the properties one at a time for this material combination.

### 3.5. Linear Response of the Stress to the Thermal Expansion Mismatch

**Figure 10:**
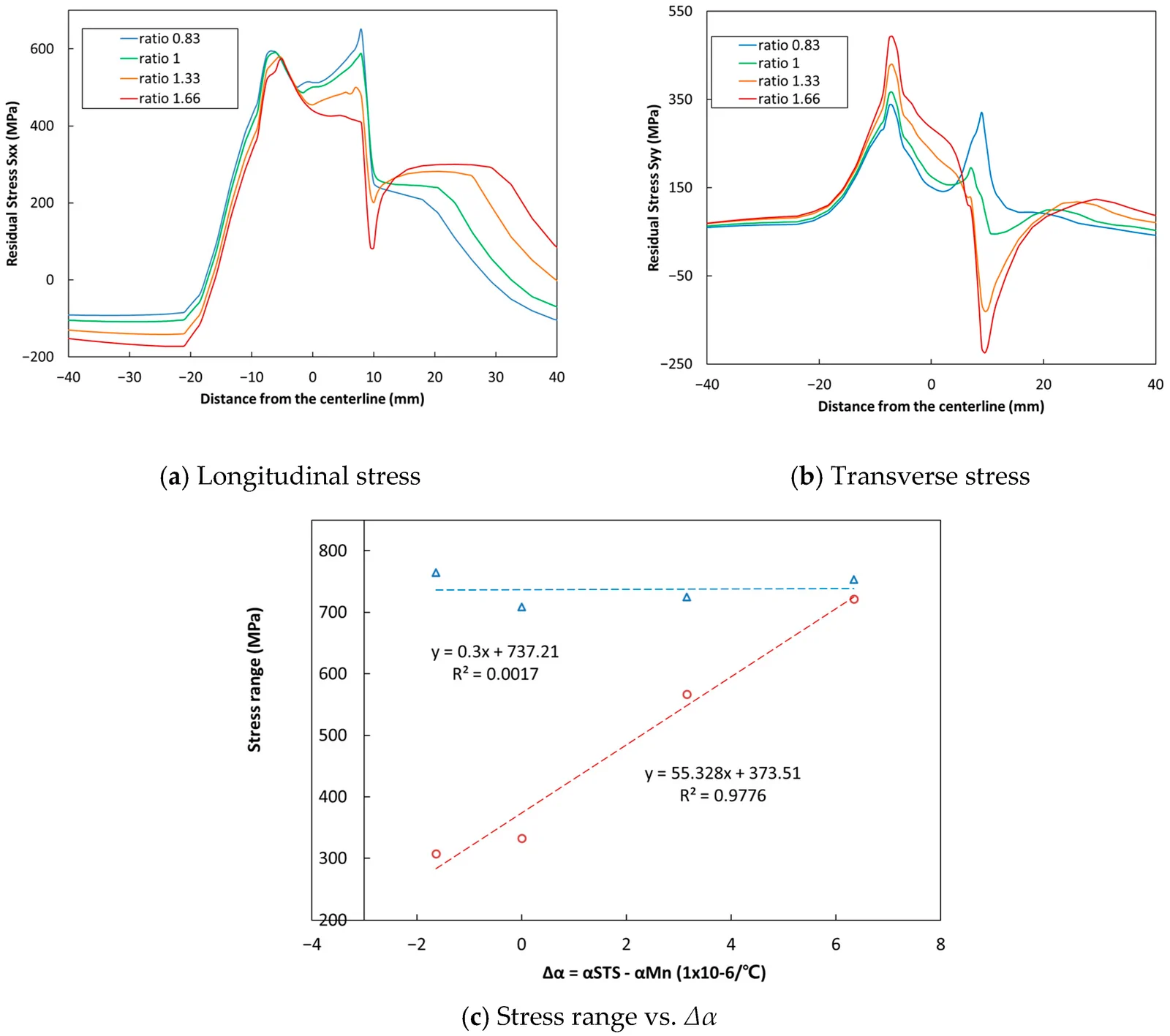
Effect of the thermal expansion coefficient ratio on residual stress: (**a**) longitudinal stress *σ*_L_, (**b**) transverse stress *σ*_T_, (**c**) linear regression of the longitudinal and transverse stress ranges against the thermal expansion mismatch *Δα* (Equations (6) and (7)). In (**c**), the triangles and the dashed blue line are the longitudinal stress range and the circles and the dashed red line the transverse stress range.

Since the dominance of the coefficient of thermal expansion has been established, the relationship between the magnitude of the mismatch and the residual stress was examined next. Analyses were carried out with the *α*(T) curve on the STS304 side, scaled so that its value became 0.83, 1.00, 1.33 and 1.66 times that of the high-manganese steel over the whole temperature range, with the value of 1.66 corresponding to the actual ratio of the two steels. The results are shown in Figure 10 and Table 11.

The behaviours of the two stress components contrast clearly here as well. While *Δα* changes from −1.63 to +6.35 × 10^−6^/°C, the range of the longitudinal stress remains between 709.1 and 764.5 MPa and shows no clear trend. The linear regression is as follows:

(6) $$\sigma_{L,\;range}\;=\;0.30\;\Delta \alpha \;+\;737.2$$

The range of the transverse stress, by contrast, increases in linear proportion to *Δα*, and the minimum stress at the interface follows the same trend.

(7) $$\sigma_{T,\;range}=55.32\;\Delta \alpha +373.5$$

(8) $$\sigma_{T,\;min}=-35.66\;\Delta \alpha -5.6$$

In Equations (6)–(8), *Δα* denotes the numerical value of the mismatch expressed in units of 10^−6^ K^−1^, so the slopes carry the units MPa/(10^−6^ K^−1^). The coefficients of determination are 0.002 for Equation (6), 0.978 for Equation (7) and 0.950 for Equation (8). Figure 10c shows only Equations (6) and (7), the regressions of the stress ranges of the two components; Equation (8) was obtained by applying the same regression to the four values of the *σ*_T_ minimum in Table 11. In other words, each increase of 1 × 10^−6^/°C in the difference of the coefficient of thermal expansion increases the transverse stress range by about 55 MPa and the compressive stress at the interface by about 36 MPa, whereas the longitudinal stress range shows no correlation with the thermal expansion mismatch.

When the coefficients of thermal expansion are equal (*α*_S_/*α*_M_ = 1.00), the minimum stress is +33.8 MPa and no compression occurs; the sign reverses to −137.2 MPa once the ratio reaches 1.33. This suggests that the interfacial compressive zone forms only when the thermal expansion mismatch exceeds a certain level. Equations (7) and (8) can be used at the design stage of a dissimilar joint to estimate the approximate magnitude of the interfacial stress gradient and of the compressive zone from the combination of the coefficients of thermal expansion of the two base metals or of the weld metal. Such a quantitative relationship linking the property mismatch to the stress was not obtained in the earlier study, in which the properties were substituted discretely [13], and it is in this respect that this study goes a step beyond the identification of the governing factor. The regression is, however, based on analyses at four levels, so the range of application of the relationships is limited to the interval of *Δα* from −1.63 to +6.35 × 10^−6^/°C and to the plate thickness and restraint conditions of this study.

### 3.6. Component-Dependent Response Caused by the Difference in Plastic Restraint

The results of Section 3.4 and Section 3.5 share a common feature. The longitudinal stress hardly responds to property substitution or to a change in the thermal expansion mismatch, whereas the transverse stress responds clearly and linearly. This is explained by the different restraint conditions experienced by the two components.

During welding, the molten zone and its vicinity are strongly restrained in the direction of the weld line by the surrounding base metal. The longitudinal stress that arises as contraction is suppressed during cooling reaches the limit of the plastic resistance of the weld material, and any further stress cannot be stored elastically but is absorbed by plastic deformation. The magnitude of the longitudinal stress is therefore bounded above by the yield strength and the work-hardening behaviour, and there is no room for a larger thermal expansion mismatch to be recorded as stress. In fact, the maximum *σ*_L_ of the homogeneous joint, 657.2 MPa, lies between the yield strength of the high-manganese steel (442 MPa) and its stress at a strain of 0.15 (953 MPa), indicating that the weld has passed yielding and entered the work-hardening regime. This is why, in Table 11, the longitudinal stress range stays between 709 and 765 MPa while *Δα* changes almost fourfold. Because the weld is in a multiaxial stress state, the comparison with the uniaxial yield strength is indicative rather than conclusive; the term used here should be understood as denoting stronger longitudinal restraint and more extensive plastic deformation rather than a strictly saturated state.

In the transverse direction, the restraint is relatively weak, and the stress does not reach the plastic limit. The difference in the amount of contraction of the two base metals therefore appears directly as elastic stress and responds linearly to the thermal expansion mismatch. At the interface, the high-manganese steel side contracts less because of its smaller coefficient of thermal expansion while the STS304 side contracts more, so a misfit of deformation develops across the interface during cooling. This misfit forms the local compressive zone, whose magnitude is proportional to the mismatch according to Equation (8).

The mechanical conditions at the interface also contribute to the divergence of the two responses. What equilibrium requires to be continuous across the interface is the traction vector t = *σ*·n, where n is the local normal of the fusion boundary, and not any single global stress component. Because the fusion boundary of a 60° groove is an inclined surface, n has components in both the transverse and the thickness directions, so the transverse stress *σ*_T_ is largely, though not exclusively, governed by the traction condition. *σ*_T_ is therefore strongly restricted from jumping in value at the interface, and the difference in the contraction of the two materials appears mainly as a steep gradient over a narrow region and as a local compressive zone. The fact that the minimum of *σ*_T_ is observed not as a step but as a local minimum of −228.4 MPa at the interface is consistent with this condition.

The longitudinal stress, *σ*_L_, which is parallel to the interface, is, by contrast, not restricted by the traction condition. The two materials on either side of the interface share the longitudinal strain through the compatibility condition, but their elastic moduli and coefficients of thermal expansion differ, so in principle *σ*_L_ could take different values at the interface. That *σ*_L_ nevertheless does not respond to the thermal expansion mismatch in the present analysis is because, as discussed above, the strong restraint in the direction of the weld line has already brought *σ*_L_ to the limit of plastic resistance, so that the stress difference that the property difference would create is absorbed by plastic deformation. In short, *σ*_T_ responds by a gradient because a jump is forbidden, whereas *σ*_L_ is permitted to jump but cannot respond because of plastic saturation.

This result answers the question of which indicator should be used to assess a dissimilar joint. If a dissimilar weld is assessed by the peak residual stress alone, the conclusion is that it is not distinguishable from a homogeneous weld within the present validation uncertainty (Table 7), because the longitudinal component is plastically saturated and is therefore an indicator insensitive to dissimilarity. The report by An et al. [6], who measured the peak residual stresses of high-manganese steel, 9% Ni steel and stainless steel joints and found them to be close to the yield strength of each material with no significant difference between homogeneous and dissimilar joints, is explained in the same way. Because the characteristic features of a dissimilar joint appear in the transverse component and in the stress gradient at the interface, these indicators should be examined together in a safety assessment.

### 3.7. Relaxation of the Interfacial Stress Under Service Loading and Implications for Integrity Assessment

We examined how the stress gradient at the interface changes when a representative monotonic tensile load is applied in the presence of welding residual stress. The applied stress of 212 MPa corresponds to 80% of the yield strength of the STS304 base metal and is not derived from a specific design load of an LNG facility. The analyses in this section were also carried out with a model separate from that of Section 3.2, so the results are presented not as a direct comparison of absolute values but as a relative change with respect to the unloaded state of each joint.

**Figure 11:**
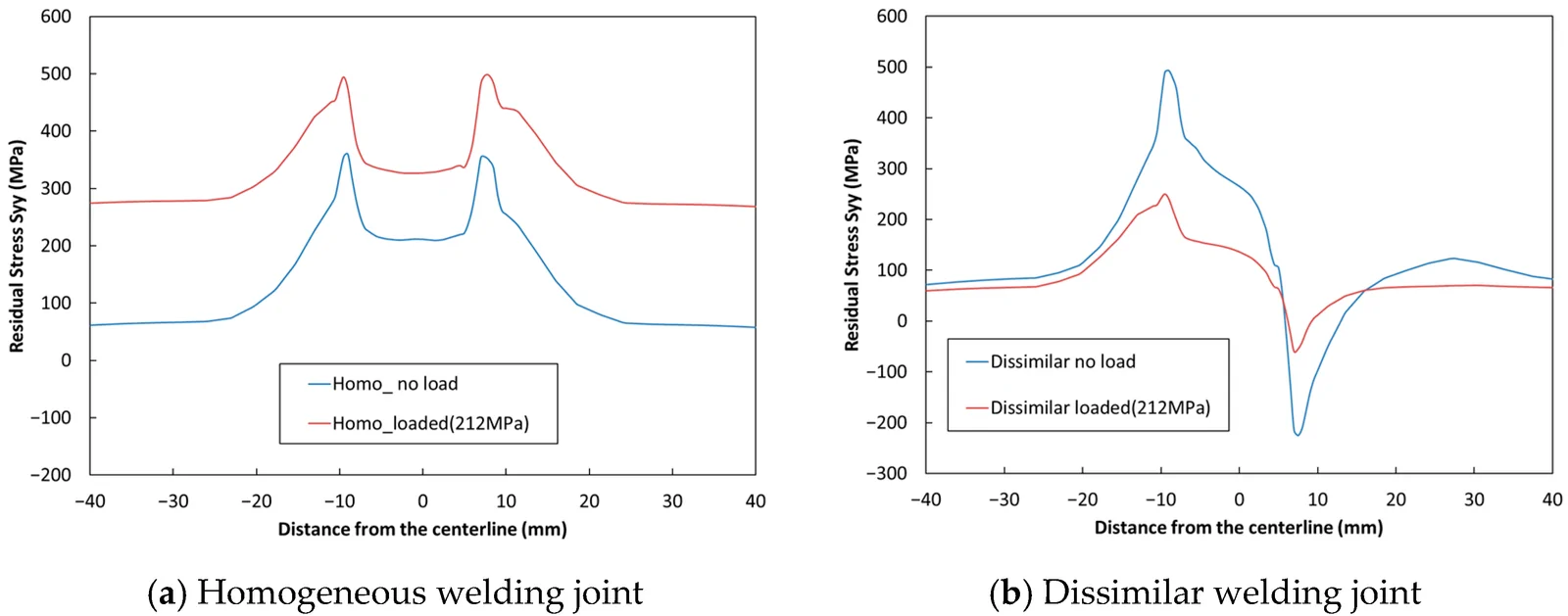
Redistribution of residual stress under a tensile load (top surface): (**a**) homogeneous joint, (**b**) dissimilar joint.

The variation of the transverse stress near the interface (over the interval 2–22 mm from the weld centre) was defined as the interfacial stress range, and its change before and after loading is given in Table 12. The stress distributions are shown in Figure 11.

In the dissimilar joint, the interfacial stress range decreased by 61% on the top surface and by 62% on the bottom surface. The compressive stress at the interface was also greatly relaxed, from −228.4 MPa to −60.4 MPa. In the homogeneous joint, by contrast, the reduction was only 22%.

This difference arises from the nature of the interfacial stress. As discussed in Section 3.6, the steep gradient at the interface is an elastic misfit caused by the difference in the contraction of the two base metals, so the misfit can be partly resolved by plastic deformation under an external load. The stress distribution of the homogeneous joint, on the other hand, is already close to plastic saturation, so there is little scope for change under load.

The interfacial stress gradient of a dissimilar joint is therefore largest in the as-fabricated state and is reduced to a considerable extent while the load is applied. The values in Table 12 are the total stresses under the applied load. The behaviour under cyclic loading lies outside the scope of this study.

## 4. Conclusions

Three-dimensional thermal elastic–plastic finite element analysis and sectioning measurements were carried out on dissimilar butt welds between high-manganese steel and STS304, and the governing factors were identified by single-property substitution. Because both steels are stable austenite, the effect of the thermal expansion mismatch is isolated without the disturbance of solid-state transformation, and the residual stress is treated in two separate components. The principal results are as follows.

(1) The thermal analysis was verified against measured temperature histories and macrographs. In the cooling period that governs the residual stress, the differences at the toe and at 3 mm were 0.5% and 0.1%, respectively; the peak temperature at the toe is an input and not an object of verification. The heat-affected zone on the high-manganese steel side is about 1.5 times wider owing to the low thermal conductivity, and the analysis reproduced this width. Against the sectioning measurements, the RMSE was 103.3 MPa for the homogeneous series and 127.0 MPa for the dissimilar series.

(2) The characteristic feature of a dissimilar joint lies not in the peak stress but in the steep gradient and the local compressive zone at the weld metal–STS304 interface 8.5 mm from the weld centre. The longitudinal range differs by 1.6% and the maximum by 77.0 MPa within the analysis–measurement deviation, whereas the transverse range increases by 51.6% and its minimum reverses from +39.2 MPa to −228.4 MPa. The compressive peak and the maximum gradient are computed quantities that the sectioning measurements cannot resolve directly, so the absolute values carry an uncertainty of the order of 100 MPa and the results that follow are relative comparisons.

(3) This interfacial stress gradient is unrelated to the welding process and to the strength mismatch of the weld metal. When the yield strength of the weld metal was increased by 50%, from 353.6 MPa to 530.4 MPa, the maximum stress rose by 33.0%, but the minimum stress changed by only 18.0 MPa and no transition to compressive stress occurred at any level.

(4) Of the four properties examined, the coefficient of thermal expansion gave a reproduction ratio of 30.6–98.1% with a mean of 57.9%, against 30.7% for the other three combined, and it alone reproduced the sign reversal of the interfacial compression. The elastic modulus gives +47.2% for the longitudinal component but −22.7% for the transverse component, so it cannot be a governing factor. Simultaneous substitution gives 102.4%, exceeding the sum of the individual contributions by 13.8 percentage points along the dissimilarity direction. The work-hardening curve, Poisson’s ratio and the heat capacity were not substituted.

(5) With respect to the mismatch *Δα*, the transverse stress range follows 55.32*Δα* + 373.5 (R^2^ = 0.978) and the interfacial compression −35.66*Δα* − 5.6 (R^2^ = 0.950), so each increase of 1 × 10^−6^/°C raises the range by about 55 MPa and the compression by about 36 MPa. The longitudinal range is uncorrelated with *Δα* (R^2^ = 0.002).

(6) The opposite responses follow from the difference in plastic restraint. Strong restraint along the weld line has already brought the longitudinal stress to extensive plastic deformation, so it cannot store the mismatch elastically, whereas the weaker transverse restraint lets the difference in contraction appear directly as stress. The interface condition acts in the same direction: continuity of the traction vector t = *σ*·n across the inclined fusion boundary restricts a jump in the transverse stress, so the mismatch appears as a steep gradient and a local compressive zone rather than as a step change.

(7) Under a tensile load of 212 MPa, 80% of the STS304 yield strength, the interfacial stress range of the dissimilar joint decreased by 61% on the top surface and 62% on the bottom surface, against 22% for the homogeneous joint, because the interfacial stress is an elastic misfit that is partly resolved by plastic deformation. The values compared are total stresses under the applied load.

## Figures and Tables

**Table 1:** Chemical compositions of the base metals (wt %).

Material	C	Si	Mn	Ni	P	S	Cr
High-Mn steel	0.35–0.55	0.10–0.50	22.5–25.5	–	≤0.03	≤0.01	–
STS304	0.016	0.21	1.72	8.26	0.029	0.020	19.25

**Table 2:** Room-temperature properties applied in the analysis.

Property	High-Mn Steel	Weld Metal	STS304
Density ρ (kg/m^3^)	7660	7660	7900
Elastic modulus E (GPa)	175	175	198.5
Yield strength *σ*_Y_ (MPa)	442	353.6	265
Stress at 0.15 strain (MPa)	953	580	318
Poisson’s ratio *ν*	0.27	0.27	0.30
Thermal expansion coefficient *α* at 20 °C (10^−6^/°C)	9.61	9.61	15.96
Thermal conductivity k (W/m·°C)	12.2	12.2	14.6
Specific heat c (J/kg·°C)	473	473	462

**Table 3:** Welding conditions.

Item	FCAW	SAW	GTAW
Consumable	PT-400M	PC-400M	T-400M
Wire diameter (mm)	1.2	4.0	2.0
Specimen size (mm)	248 × 302	496 × 445	248 × 297
Groove angle	60°	60°	60°
Root gap (mm)	6	4	–
Number of passes	12	3	11
Root pass	140 A–28 V, 12 cm/min	500 A–30 V, 30 cm/min	235 A–15 V, 16 cm/min
Filling pass	180 A–32 V, 23 cm/min	500 A–30 V, 26 cm/min	220 A–15 V, 10 cm/min
Heat input (filling, kJ/cm)	15.0	34.6	19.8
Shielding gas/flux	CO_2_ 25 L/min	POS-CF1 (flux)	Ar 25 L/min
Interpass temperature (°C)	≤150	≤150	≤150

**Table 4:** Analysis cases.

Group	Case	Purpose	Results
A	High-Mn/High-Mn homogeneous and High-Mn/STS304 dissimilar (FCAW)	Reference and target behaviour	3.1, 3.2
B	Three welding processes (FCAW/SAW/GTAW)	Effect of process	3.3
B	Weld metal yield strength 353.6/397.8/442.0/530.4 MPa	Effect of strength mismatch	3.3
C	*σ*_Y_ → 265 MPa	Contribution of yield strength	3.4
C	E → 198.5 GPa	Contribution of elastic modulus	3.4
C	k → 14.6 W/m·°C	Contribution of thermal conductivity	3.4
C	*α* → 15.96 × 10^−6^/°C	Contribution of thermal expansion	3.4
C	Simultaneous substitution of all four properties	Check of additivity	3.4
D	*α*_S_/*α*_M_ = 0.83/1.00/1.33/1.66	Derivation of quantitative relation	3.5
E	Tensile load of 212 MPa	Behaviour under service condition	3.7

**Table 5:** Comparison of measured and calculated temperature histories.

Location	Largest Recorded Value (°C)	Calculated Peak (°C)	RMSE (°C)	At 40.7 s, Meas./Calc. (°C)
Toe (8 mm)	1573.3 *	1600.0 **	121.5	338.1/339.7
11 mm	624.1	683.1	85.5	303.7/303.5
20 mm	185.7	(140.1)	42.9	185.5/140.1
30 mm	108.5	(88.6)	9.6	80.1/88.6

* Indication only. The value lies above the specified upper limit of 1260 °C for a K-type thermocouple, beyond which the electromotive force–temperature relation is not guaranteed; it was not used in the verification. ** Input value: the activation temperature assigned to the weld metal elements, not a prediction; the RMSE of this row covers 13.9–40.7 s. Values in parentheses are those at 40.7 s and are not peak values.

**Table 6:** Comparison of measured and calculated residual stresses.

Joint Series	Component	Number of Points	RMSE (MPa)	Mean Deviation (MPa)	Maximum Deviation (MPa)
Homogeneous (High-Mn/High-Mn, FCAW)	*σ*_L_	17	113.1	+64.8	218.3
Homogeneous (High-Mn/High-Mn, FCAW)	*σ*_T_	11	86.0	−51.7	151.9
Homogeneous (High-Mn/High-Mn, FCAW)	All	28	103.3	+19.0	218.3
Dissimilar (High-Mn/STS304, 3 processes)	*σ*_L_	92	124.4	+10.9	438.5
Dissimilar (High-Mn/STS304, 3 processes)	*σ*_T_	33	134.0	−2.9	289.6
Dissimilar (High-Mn/STS304, 3 processes)	All	125	127.0	+7.3	438.5

**Table 7:** Residual stresses of the homogeneous and dissimilar joints (top surface).

Component	Item	Homogeneous	Dissimilar	Change
*σ*_L_	Maximum (MPa)	657.2	580.2	−11.7%
*σ*_L_	Minimum (MPa)	−84.0	−172.8	–
*σ*_L_	Range (MPa)	741.2	753.0	+1.6%
*σ*_L_	Maximum gradient (MPa/mm)	120.1	265.4	+121%
*σ*_T_	Maximum (MPa)	515.0	493.2	−4.2%
*σ*_T_	Minimum (MPa)	+39.2	−228.4	Sign reversal
*σ*_T_	Range (MPa)	475.9	721.6	+51.6%
*σ*_T_	Maximum gradient (MPa/mm)	134.5	195.1	+45%

**Table 8:** Effect of the yield strength of the weld metal (FCAW; homogeneous high-manganese steel joint, separate model from Section 3.2).

Weld Metal Yield Strength (MPa)	Relative to Base Metal	*σ*_T_ Maximum (MPa)	*σ*_T_ Minimum (MPa)
353.6	−20%	571.1	49.0
397.8	−10%	611.1	48.6
442.0	0%	656.5	48.0
530.4	+20%	759.6	67.0

**Table 9:** Reproduction ratio R for each material property factor (%).

Substituted Factor	*σ*_L_ Top	*σ*_L_ Bottom	*σ*_T_ Top	*σ*_T_ Bottom	Mean
Yield strength *σ*_Y_	21.3	18.1	13.7	10.8	16.0
Elastic modulus E	47.2	0.3	−22.7	1.5	6.6
Thermal conductivity k	1.7	1.4	2.0	27.4	8.1
Thermal expansion coefficient *α*	30.6	44.3	58.6	98.1	57.9
All four simultaneously	92.2	94.5	105.1	118.0	102.4

**Table 10:** Minimum transverse stress (top surface).

Case	Homogeneous	Dissimilar	*σ*_Y_ Subst.	E Subst.	k Subst.	*α* Subst.	All Four
*σ*_T_ minimum (MPa)	+39.2	−228.4	+36.3	+43.3	+45.8	−88.8	−276.7

**Table 11:** Residual stress for each thermal expansion coefficient ratio (top surface). The ratio *α*_S_/*α*_M_ and *Δα* refer to the room-temperature values; the entire *α*(T) curve on the STS304 side was scaled by the corresponding factor.

*α*_S_/*α*_M_	*α*_S_ (10^−6^/°C)	*Δα* (10^−6^/°C)	*σ*_L_ Range (MPa)	*σ*_T_ Range (MPa)	*σ*_T_ Minimum (MPa)
0.83	7.98	−1.63	764.5	307.9	+29.0
1.00	9.61	0.00	709.1	332.9	+33.8
1.33	12.77	+3.15	724.6	566.9	−137.2
1.66	15.96	+6.35	753.0	721.6	−228.4

**Table 12:** Change of the interfacial stress range under a tensile load.

Joint	Surface	Unloaded (MPa)	Loaded (MPa)	Reduction
Dissimilar	Top	462.4	178.9	−61%
Dissimilar	Bottom	542.4	204.4	−62%
Homogeneous	Top	279.1	218.0	−22%

## Data Availability

The temperature history data supporting the verification in Section 3.1 are available upon request from the corresponding author. The temperature-dependent property data of the high-manganese steel are not publicly available owing to restrictions imposed by the material supplier.

## Abbreviations

The following abbreviations are used in this manuscript:

FCAW	Flux-cored arc welding
FE	Finite element
GTAW	Gas tungsten arc welding
LNG	Liquefied natural gas
RMSE	Root mean square error
SAW	Submerged arc welding
TWIP	Twinning-induced plasticity

## References

[1] Sun Z., Karppi R. (1996). The application of electron beam welding for the joining of dissimilar metals: An overview. J. Mater. Process. Technol..

[2] Deng D., Kiyoshima S., Ogawa K., Yanagida N., Saito K. (2011). Predicting welding residual stresses in a dissimilar metal girth welded pipe using 3D finite element model with a simplified heat source. Nucl. Eng. Des..

[3] Han I.W., Lee B.K., Park S.H., Kang C.Y. (2017). Microstructure and mechanical properties of cryogenic high-manganese steel weld metal. Int. J. Offshore Polar Eng..

[4] International Maritime Organization (2018). Interim Guidelines on the Application of High Manganese Austenitic Steel for Cryogenic Service.

[5] An G., Hong S., Park J., Ro C., Han I. (2017). Identification of correlation between fracture toughness parameters of cryogenic steel weld joints. J. Weld. Join..

[6] An G., Park J., Lim W., Park H., Han I. (2022). Characteristics of welding residual stress distribution in dissimilar weld joints. Metals.

[7] Leggatt R.H. (2008). Residual stresses in welded structures. Int. J. Press. Vessels Pip..

[8] Sędek P., Brózda J., Wang L., Withers P.J. (2003). Residual stress relief in MAG welded joints of dissimilar steels. Int. J. Press. Vessels Pip..

[9] Lee C.-H., Chang K.-H., Park J.-U. (2013). Three-dimensional finite element analysis of residual stresses in dissimilar steel pipe welds. Nucl. Eng. Des..

[10] Ranjbarnodeh E., Serajzadeh S., Kokabi A.H., Fischer A. (2011). Effect of welding parameters on residual stresses in dissimilar joint of stainless steel to carbon steel. J. Mater. Sci..

[11] Katsareas D.E., Youtsos A.G. (2005). Residual stress prediction in dissimilar metal weld pipe joints using the finite element method. Residual Stresses VII, ICRS7.

[12] Akbari D., Sattari-Far I. (2009). Effect of the welding heat input on residual stresses in butt-welds of dissimilar pipe joints. Int. J. Press. Vessels Pip..

[13] Eisazadeh H., Achuthan A., Goldak J.A., Aidun D.K. (2015). Effect of material properties and mechanical tensioning load on residual stress formation in GTA 304-A36 dissimilar weld. J. Mater. Process. Technol..

[14] Lee C.-H., Chang K.-H. (2011). Prediction of residual stresses in high strength carbon steel pipe weld considering solid-state phase transformation effects. Comput. Struct..

[15] Deng D., Luo Y., Serizawa H., Shibahara M., Murakawa H. (2003). Numerical simulation of residual stress and deformation considering phase transformation effects (Mechanics, Strength & Structural Design). Trans. JWRI.

[16] Li S., Li J., Sun G., Deng D. (2023). Modeling of welding residual stress in a dissimilar metal butt-welded joint between P92 ferritic steel and SUS304 austenitic stainless steel. J. Mater. Res. Technol..

[17] Lee M.-S., Seo J.-K. (2021). Residual stress study of high manganese steel riser pipe manufactured by longitudinal butt welding (1): Residual stress measurement and FE analysis. J. Weld. Join..

[18] Lee M.-S., Seo J.-K. (2021). Residual stress study of high manganese steel riser pipe manufactured by longitudinal butt welding (2): Equation for predicting welding induced residual stresses established through parametric study. J. Weld. Join..

[19] Park J., An G., Kim D. (2022). Residual stress and deformation characteristics by FSW and SAW of wear resistant steel. J. Weld. Join..

[20] Lee C.-H., Chang K.-H. (2007). Numerical analysis of residual stresses in welds of similar or dissimilar steel weldments under superimposed tensile loads. Comput. Mater. Sci..

[21] Bang H.-S., Bang H.-S., Kim Y.-C., Joo S.-M., Joa S.-W., Ro C.-S. (2009). Influence of welding residual stress on the mechanical behavior of externally loaded dissimilar SS400-STS304 steel weldment. J. Weld. Join..

[22] Deng D., Murakawa H. (2006). Numerical simulation of temperature field and residual stress in multi-pass welds in stainless steel pipe and comparison with experimental measurements. Comput. Mater. Sci..

[23] Goldak J.A., Chakravarti A., Bibby M. (1984). A new finite element model for welding heat sources. Metall. Trans. B.

[24] DuPont J.N., Marder A.R. (1995). Thermal efficiency of arc welding processes. Weld. J..

[25] Stenbacka N. (2013). On arc efficiency in gas tungsten arc welding. Soldag. Insp..

[26] Henning C.D., Parker R. (1967). Transient response of an intrinsic thermocouple. J. Heat Transf..

[27] Gat U., Kammer D.S., Hahn O.J. (1975). The effect of temperature dependent properties on transients measurement with intrinsic thermocouple. Int. J. Heat Mass Transf..

[28] Attarha M.J., Sattari-Far I. (2011). Study on welding temperature distribution in thin welded plates through experimental measurements and finite element simulation. J. Mater. Process. Technol..

